# Defining the Contribution of *CNTNAP2* to Autism Susceptibility

**DOI:** 10.1371/journal.pone.0077906

**Published:** 2013-10-17

**Authors:** Srirangan Sampath, Shambu Bhat, Simone Gupta, Ashley O’Connor, Andrew B. West, Dan E. Arking, Aravinda Chakravarti

**Affiliations:** 1 Center for Complex Disease Genomics, McKusick-Nathans Institute of Genetics Medicine, Johns Hopkins University School of Medicine, Baltimore, Maryland, United States of America; 2 Department of Neurology and Neurobiology, Center for Neurodegeneration and Experimental Therapeutics, University of Alabama at Birmingham, Birmingham, Alabama, United States of America; Children's National Medical Center, Washington, United States of America

## Abstract

Multiple lines of genetic evidence suggest a role for *CNTNAP2* in autism. To assess its population impact we studied 2148 common single nucleotide polymorphisms (SNPs) using transmission disequilibrium test (TDT) across the entire ~3.3 Mb *CNTNAP2* locus in 186 (408 trios) multiplex and 323 simplex families with autistic spectrum disorder (ASD). This analysis yielded two SNPs with nominal statistical significance (rs17170073, *p* = 2.0 x 10^-4^; rs2215798, *p* = 1.6 x 10^-4^) that did not survive multiple testing. In a combined analysis of all families, two highly correlated (*r* ^2^ = 0.99) SNPs in intron 14 showed significant association with autism (rs2710093, *p* = 9.0 x 10^-6^; rs2253031, *p* = 2.5 x 10^-5^). To validate these findings and associations at SNPs from previous autism studies (rs7794745, rs2710102 and rs17236239) we genotyped 2051 additional families (572 multiplex and 1479 simplex). None of these variants were significantly associated with ASD after corrections for multiple testing. The analysis of Mendelian errors within each family did not indicate any segregating deletions. Nevertheless, a study of *CNTNAP2* gene expression in brains of autistic patients and of normal controls, demonstrated altered expression in a subset of patients (*p* = 1.9 x10^-5^). Consequently, this study suggests that although *CNTNAP2* dysregulation plays a role in some cases, its population contribution to autism susceptibility is limited.

## Introduction

Autism (MIM 209850) is a highly heritable, early onset childhood neuropsychiatric developmental disorder characterized by impairments in the three core domains of social interaction and communication, language development, and repetitive restricted behavior and interests. The phenotype occurs within a wide range constituting the autistic spectrum disorders (ASD) that includes Asperger’s syndrome, childhood disintegrative disorder and pervasive developmental disorder not otherwise specified; autism is the most severe and archetype of the ASD. Among children within the ages of 4-6 years, the prevalence of autism is 20 per 10,000 live births and for ASD it is 60 per 10,000 live births [1]. Males are more frequently affected with autism than females (sex ratio ~4.2:1) [2]. The substantial increase in the prevalence of ASD in the last two decades presents an enormous public health challenge with great societal and individual consequences [3,4]. 

The clear evidence for an increased prevalence of autism among siblings of autistic probands, and the greater disease concordance rate among monozygous than dizygous twins, shows a significant genetic influence in autism etiology with heritability estimates of 80-90% [5-7]. Nevertheless, considering that autism represents one of the most heritable neuropsychiatric disorders, progress in identifying the molecular basis of autism has been sluggish, and its pathophysiology remains unclear. Over the last decade, numerous gene discovery studies have implicated a small number of genes with either rare highly-penetrant mutations, or low-penetrant common variants or copy-number variants (CNV) that together explain no more than 15-30% of the population prevalence [8-12]. Although these studies have implicated many genes of diverse function, the underlying genetic heterogeneity ensures that no single gene accounts for the majority of autism. Despite such heterogeneity several chromosomal loci have been shown to be linked to ASD by multiple independent linkage and association studies, including those at 2q24-2q31[13-15], 7q22-7q31 [16-23], 7q34-7q36 [24,25] and 17q11-17q21 [26-30]. Recurrent structural chromosomal changes involving deletions and duplication at 7q11, 15q11-15q13, 17p11.2, 22q11.2 and 22q13 have also been associated with syndromic forms of autism [31]. 

*CNTNAP2*, or contactin associated protein-like 2, is one of the genes with the strongest evidence of autism susceptibility with convergent evidence from several independent studies. In fact, mice lacking *CNTNAP2* show striking similarity to the core deficits of behavioral and cognitive functions that are seen in patients with ASD signifying its vital role in brain development [32]. This gene was first identified in autism by linkage in a Old Order Amish family with an autosomal recessive founder null mutation leading to cortical dysplasia-focal epilepsy (CDFE; MIM 610042) characterized by the presence of neuronal migration abnormalities, seizures, intellectual disability, language regression, hyperactivity, impulsive and aggressive behavior, and ASD [33]. Subsequently, several independent studies using whole genome linkage, association and CNV analysis have demonstrated the role of rare [34], common [35-37] and deletion variants [34,38,39] at *CNTNAP2* as a susceptibility factor for idiopathic ASD and autism, and related language quantitative traits. In addition, haploinsufficiency for *CNTNAP2*, resulting from gene disruption, has been implicated in other clinically distinct behavioral disorders such as Gilles de la Tourette syndrome [40] (GTS; MIM 137580), attention-deficit/hyperactivity disorder [41] (ADHD) and, schizophrenia and epilepsy [42]. Finally, homozygous and compound-heterozygous deletions and mutations in *CNTNAP2* and *NRXN1*, both distant members of the evolutionarily conserved synaptic proteins of the neurexin superfamily, have been discovered in patients with severe mental retardation, autistic behavior, seizures and microcephaly, a phenotype resembling Pitt-Hopkins syndrome (PHS; MIM 610954) [43].

*CNTNAP2* is one of the largest mammalian genes that spans more than 3.3 Mb and maps to the common fragile site FRA7I that is associated with genomic instability in solid tumors. *CNTNAP2* encodes CASPR2 with expression restricted to neurons. CASPR2 is a transmembrane scaffolding protein belonging to the neurexin superfamily that clusters voltage-gated potassium channels at the nodes of Ranvier [44]. Consequently, *CNTNAP2* is a very large mutational target, and its restricted and early expression in the nervous system, potentially deregulated by mutations in autism, fit well with current understanding of autism pathobiology.

We, and others, have identified common non-coding variants in *CNTNAP2* that are associated with some features of autism: rs7794745 in intron 2 is associated with strict autism [36], whereas, the intron 13 variants rs2710102 and rs17236239 are associated with the quantitative autism endophenotypes of age-at-first word [35] and specific language impairment [37]. In addition, other variants within *CNTNAP2* exons 13-15 show modest association with specific language impairment [37]. Despite some degree of overlap in the families used for analyses, these genetic signals at *CNTNAP2* represent statistically significant and independent associations. Consequently, we designed a study to comprehensively search for genetic associations within the entire *CNTNAP2* locus that could contribute to autism and ASD susceptibility. 

 In this study, we perform genetic association analyses of *CNTNAP2* with four major improvements. First, we perform fine-mapping of the entire 3.3 Mb *CNTNAP2* locus at high marker density by genotyping 2148 common SNPs using a custom genotyping array and at high accuracy. Second, we examine a broad segment of the underlying liability by including both multiplex and simplex families, and both autism and ASD probands. Third, we use the family-based transmission disequilibrium test (TDT) to minimize population stratification. Fourth, we examine a large sample size of families (731 probands). An independent source of evidence of *CNTNAP2*’s role was obtained by examining its gene expression in the occipital cortex. Using a logistic expression analysis, we observed that altered *CNTNAP2* expression is highly associated with autism status (*p* = 1.9 x 10^-5^). Although we have demonstrated dysregulation of *CNTNAP2* gene expression, it appears that common variants in *CNTNAP2* have a limited role in autism susceptibility. It remains entirely possible that *CNTNAP2* harbors a diversity of coding and non-coding mutations that will be important for understanding autism and ASD pathophysiology, but its epidemiological impact is small.

## Materials and Methods

### Study Samples

The multiplex families used in this study came from the Autism Genetic Resource Exchange (AGRE, http://research.agre.org/) [45] and the National Institute of Mental Health Autism Genetics Initiative repositories (NIMH, https://www.nimhgenetics.org/available_data/autism/). The majority of the families self-identify themselves as being ‘white’ (75.1%); the remainders are African-American (1.5%), Asian (2.4%), Hispanic (5.3%) and of mixed ancestry (15.7%). The simplex families were derived from the Simons Foundation Autism Research Initiative (SFARI) Simplex Collection (SSC) [46]. The self-reported race/ethnicities of these samples were: white (75.2%), African-American (4.7%), Asian (3.7%), Hispanic (3.0%) and mixed ancestry (13.4%). The ADI-R and ADOS instruments were available for only 68% and 52% of the affected subjects in this study, respectively. We excluded families with known chromosomal anomalies and four families with >10% Mendelian segregation errors. 

### Brain tissue

 Frozen brain tissue samples from the cerebral cortex (BA 19) of 39 autism cases and 44 control subjects were obtained via the Autism Tissue Program (http://www.atpportal.org) that includes the Harvard Brain Tissue Resource center, the NICHD Brain and Tissue Bank at the University of Maryland and the University of California San Diego. 

### SNP selection and genotyping

We designed a custom, targeted genotyping array (TG-array) containing 3150 SNPs that spanned the 3.3 Mb *CNTNAP2* locus (hg18; 144 751 846-148 054 557), with a median spacing between SNPs of 663 bp, using Affymetrix technology (GeneChip Custom 3K SNP Kit). The SNPs were selected from the HapMap Phase II [47] data (r22, NCBI build 36) from the European (CEU), African (YRI) and Asian (CHB-JPT) samples. This consisted of tagging SNPs selected using the Haploview Tagger software [48] with settings of *r* ^2^ = 0.70 and minor allele frequency (MAF) >0.05 across all populations. We augmented this list by filling in physical gaps with tag SNPs that had MAF >0.01 and *r* ^2^ = 0.95. Of the 3900 SNPs we identified, 3150 met Affymetrix manufacturing criteria and were on the array (TG-array). Of the three known common variants at *CNTNAP2* associated with autism and QTL phenotypes, only rs2710102 was present on the TG-array; thus, rs7794745 and rs17236239 were separately genotyped using TaqMan assays (Applied Biosystems, Foster City, CA). The genotyping assay is described in detail in Supplementary Methods section (see Information S1). 

Stage II validation studies were performed by genotyping multiplex (572 families) and simplex families (1479 trios) for six SNPs (rs7794745, rs17170073, rs2710093, rs2710102, rs2215798, rs17236239) using either TaqMan or primer extension MALDI-TOF genotyping (Autoflex HT, Sequenom) assays, except for rs2710093 which was genotyped using both TaqMan and Sequenom assays. Pre-designed assays were used for genotyping all SNPs except for rs2710093 that was custom designed. Manufacturer’s protocols were followed for all assays. Custom primer sequences for rs2710093 are provided in the Supplementary Methods section (see Information S1). 

### Genotyping quality control analysis

The overall call rate for the TG-array panel was 99.7% based on 1749 samples. In order to evaluate the reproducibility of TG-array genotypes, measured as the proportion of mismatch to the consensus genotype for each sample across all 3150 TG-array SNPs, we performed replicate genotyping of Affymetrix control (27 replicates) and nine simplex samples (at least 7 replicates each). The mean proportion of mismatch to the consensus genotype was 8.0 x 10^-4^ and 3.0 x 10^-4^ for the Affymetrix control and nine simplex samples. Ten percent of the TaqMan assay was also performed in replicate, with a non-missing concordance rate of 100%. Samples genotyped by TG-array in stage I were also genotyped by either TaqMan or Sequenom assays in stage II, and their concordance were as follows. In the simplex collection, for SNPs rs2215798 (TaqMan), rs2710102 (Sequenom) and rs2710093 (Sequenom) the concordance rates were 99.9%, 98.9% and 99.8%, respectively, based on 938 overlapping samples. In the NIMH collection, for SNPs rs17170073 (TaqMan), rs2710102 (TaqMan) and rs2710093 (TaqMan) the concordance rates were 100%, 99.9% and 99.1%, respectively, based on 768 overlapping samples. For rs2710093, that was genotyped by both TaqMan and Sequenom in stage II, the concordance was 100%, based on 5147 overlapping samples. The genotype concordance for rs7794745 from our previous study [36] and present study was 99.8% based on 3760 overlapping samples. These results indicate that the genotype data we generated are of excellent quality in both the simplex and multiplex families.

### Association analysis

Family based association analysis using the transmission disequilibrium test (TDT) was performed using PLINK [49] software (v1.07). Multiple affected sibs in multiplex families violate the independence of genotypes and can bias transmission ratios and *p*-values when using PLINK. We performed TDT analysis using FBAT [50] and found that this effect is very small owing to the large no of families used in our study (see Information S1). Hence, all TDT analyses were performed using PLINK. Four families with large Mendelian errors (>10%) suggestive of sample errors or non-paternity were removed before analysis. Before further analysis, genotype data were filtered to remove families and SNPs with call rates <90%, minor allele frequency <5%, Hardy-Weinberg equilibrium *p*-value <0.001 and Mendelian errors >5%. The genotype clusters of the SNPs with significant *p*-values were manually examined. In order to identify large deletions we analyzed families for the presence of clustered Mendelian errors. For a SNP with previous evidence for either autism risk (rs7794745) or its endophenotypes (rs2710102 and rs17236239), a three SNP test and one-sided *p*-value was used in stage II, but a genome-wide correction and two-sided *p*-value in the merged stage I and II analysis. 

### Correction for multiple testing

Given the large number of correlated SNPs tested in our study we used correlation measures based on PCA to estimate the number of independent SNPs to correct for the nominal significance threshold (α = 0.05). We used SNPSpD (see Information S1) to calculate the effective number of SNPs (M_eff_). Based on this method, we estimated the effective number of SNPs to be 663.12, giving an experiment-wise significance threshold required to keep type-I error rate of 5% as 7.5 x 10^-5^ (0.05/663.12).

### Genotype imputation

To improve the polymorphism content of the *CNTNAP2* locus we performed imputation of genotypes not experimentally obtained using BEAGLE (v3.3.0) [51] and MaCH (v1.0) [52]. The imputed genotypes were used to identify potential additional association signals and were not used to fill in missing genotypes of typed markers. Because our samples included admixed individuals, based on self-reports and by principal component analysis, all imputations were performed using combined reference haplotypes from multiple populations since this improves imputation accuracy [52,53]. Additionally, all reference haplotypes were generated to contain only an overlapping panel of SNPs from all populations. The alleles of 497 SNPs from the TG-array panel were flipped to the + strand to ensure consistent alleles between the genotyped and reference samples. Detailed method is described in the Supplementary Methods section (see Information S1). 

Imputation quality was assessed by masking a small proportion of the SNPs prior to imputation, and comparing the genotypes at imputed versus genotyped SNPs post-imputation. Of the 909 and 1687 overlapping SNPs, we masked 2.8% (26 and 47 SNPs), 3.6% (34 and 60 SNPs), and 5.7% (51 and 96 SNPs) before imputation. The genotype concordance rate varied depending on the percentage and the identity of the masked SNPs and with maximum and minimum concordance rates of 98.29% and 97.1% respectively. In addition, BEAGLE was marginally better than MaCH at imputation with far fewer Mendelian errors despite individuals being imputed as unrelated in MaCH. Hence all association analyses were performed on genotypes imputed using the BEAGLE program. 

### Population stratification tests

Principal Component Analysis (PCA) was performed on those multiplex and simplex families that had prior genome-wide SNP data using one affected individual per family. PCA was primarily performed to select individuals of uniform ancestry for association analysis. In the multiplex collection, Affymetrix 500K data were available from the NIMH Center for Collaborative Genetics for 397 out of 408 stage I samples (184/186 families), and 890 out of 1089 stage II samples (501/572 families). For the simplex collection, Illumina 1M and 1MDuo data were available for 317 out of 323 stage I samples only. 

HapMap Phase II populations of CEU, YRI, CHB + JPT (270 samples) were used as reference populations. Of the 474 310 SNPs common between HapMap II and NIMH genome-wide data, the following SNPs were filtered out: 395 SNPs with ambiguous chromosome assignment, 77 314 A/T and G/C polymorphisms, 25 monomorphic SNPs and 86 890 SNPs with >5% missing data. The remaining 309 686 SNPs from 270 HapMap II, 685 multiplex samples (184 stage I and 501 stage II), 317 (stage I) simplex samples were merged and further tested for stratification by PCA. All PCAs were performed using the default parameters in the software smartpca from the EIGENSOFT (v3.0) package [54]. The first two principal components explaining 11% of the total variance were plotted using R (v2.10.1). The HapMap reference populations clustered into three distinct clusters and population substructure of the stage I and stage II samples were interpreted by visual inspection of the projection of the top two principal components over the HapMap reference population. All analyses were repeated by using an LD pruned set (*r* ^2^<0.50) of 99 087 genome-wide SNPs with similar results. PCA indicated that in the multiplex collection, 96% (stage I) and 72% (stage II) were of European ancestry, whereas this was 77% (stage I) in the simplex collection. The rest of the families in both multiplex and simplex collections had either CHB + JPT- or YRI-related ancestry or admixtures between them. 

### Real time PCR analysis

 Total RNA was extracted from fresh frozen postmortem brain tissue using TRIzol reagent (Invitrogen, Carlsbad, CA) according to the manufacturer’s protocol. Complementary DNA (cDNA) was generated using the Superscript III First-Strand Synthesis kit (Invitrogen). cDNA was diluted 1:5 in 10 mM Tris (pH 8.5) and 1 μl of diluted cDNA was used per 20 μl PCR reaction. Quantitative real-time PCR was performed on a CFX96 thermocycler (BioRad, Hercules, CA) using iQ Powermix (Biorad) under conditions optimized for multiplex reactions. Primer and probe sets were optimized until efficiencies were between 95-105% in standard curves of control cDNA dilutions. The following highly efficient primer/probe sets were utilized: *CNTNAP2* F5’- CCTCCTTTGCCTTCTTGGTTT G-3’; *CNTNAP2* R5’- CTCCAGTTCTTCCGGGCTTG-3’; *CNTNAP2* Probe 5’-FAM- AGCCTTCGTTCTCCCTCCGTGCG–BHQ1-3’; *MAP2* F5’-TCA CGCACACCAGGCACTCC-3’; *MAP2* R5’-TCACTCGGCACCAAGATG-3’; *MAP2* Probe 5’-HEX-TCACGCACACCAGGCACTCC-BHQ1-3’; *TBP* F 5’-TTATGGCACTGGACTGACC-3’; *TBP* R 5’-CTGCTGCTGTTGCTGTTG-3’; *TBP* probe 5’-cy5- CTGCTGCTGCTGCTGCTGC-BHQ2-3’. BHQ; black hole quencher. For multiplex quantitative real time PCR, 0.5 μM each F and R primer and 0.2 μM each probe were used in 20 μL reactions. The efficiency of each primer/probe in the multiplex reaction was determined by running a 1:5 serially diluted control cDNA in parallel on every plate. Runs where primer/probe sets were outside of 100 ± 5% efficiency were excluded and performed again with adjusted cycling conditions until satisfactory efficiency was achieved. 

 For each assay, C_t_ was determined for experimental and control probes, and relative levels of expression were provided as Δ C_t_ (experimental C_t_ - control C_t_). The average and standard deviation (SD) across at least three replicate assays was calculated, and any sample with SD ≥0.5 was inspected to determine whether one of the three assays was an outlier, defined as having a C_t_ difference >1.0 from the other two assays. After outlier removal, the correlation between each of the assays was determined (all r^2^ ≥0.98), and if necessary, adjusted so that the Y-intercept intersects 0 (we note that while correlations were extremely high, absolute Δ C_t_ could be off by a constant factor, which has an impact if not all data points are available from replicate assays). Final expression levels were then calculated from the average of the standardized Δ C_t_ as 2^-ΔCt^. 

### Graphical and Statistical analyses

 All expression analyses were carried out using R (v2.10.1). Distributions of *CNTNAP2* expression were graphically analyzed, as was natural log transformed values, to assess departure from normality. Association of log transformed expression levels with possible covariates (age, age^2^, sex, PMI, site of collection) was determined in controls using linear regression models. To determine association with autism, we used logistic regression, with expression levels as the independent variable. Bayesian information content (BIC) was used to determine the significance of adding non-linear terms. 

## Results

### Transmission disequilibrium test

The frequency distribution of the custom panel of 3150 SNPs indicates that the *CNTNAP2* genomic interval was sufficiently covered with common variants (Figures S1A and S1B in Information S1), with 2215 SNPs having a MAF greater than 5%. Genotypes for 3150 SNPs spanning the *CNTNAP2* gene were filtered prior to all family-based association analyses (see Materials and Methods section). Primarily, TDT was performed on parent-offspring trios in both multiplex and simplex families independently and also by merging data from both family types, yielding independent signals of genetic effect at the *CNTNAP2* locus. 

**Figure 1:**
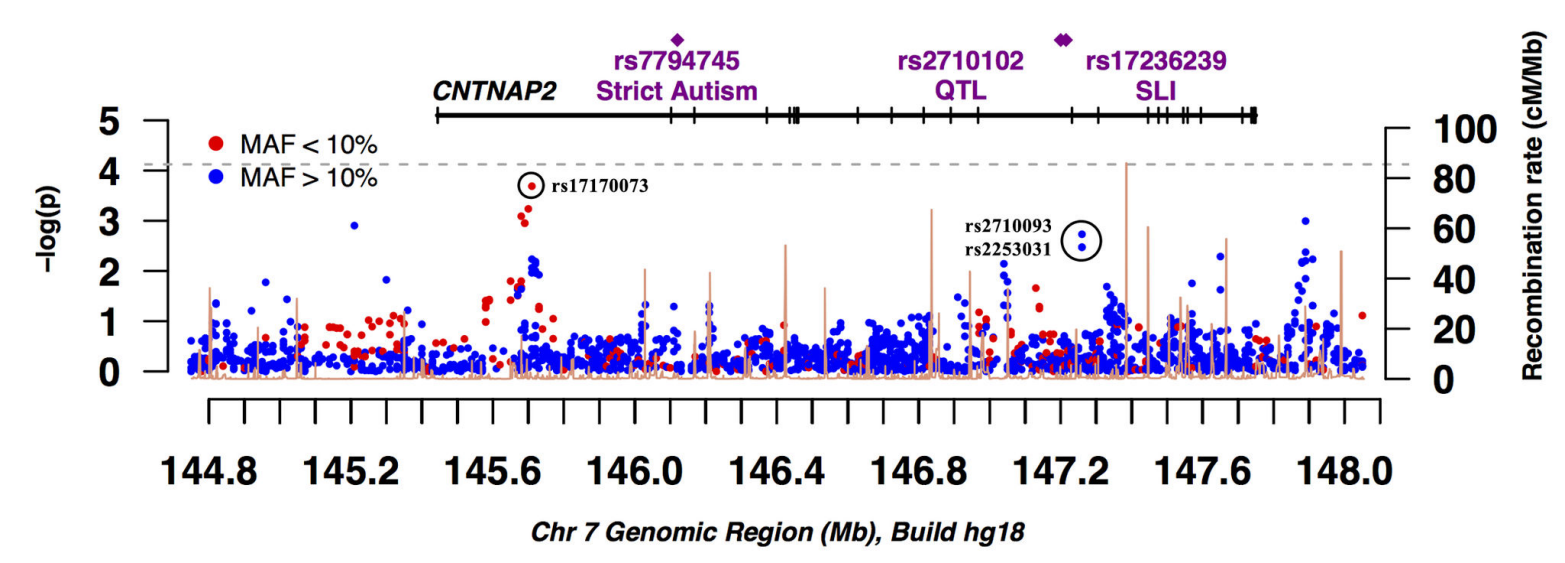
A plot of -log_10_ *p* across *CNTNAP2* from family-based association test (TDT) results on 186 multiplex families. The physical position within the *CNTNAP2* locus in megabases (Mb) and the -log_10_ *p*-values are shown on the x and y-axis, respectively. SNPs with minor allele frequency (MAF) <10% and those with MAF ≥10% are shown as red and blue filled circles, respectively. A horizontal line represents the *CNTNAP2* gene, with vertical hash marks indicating exons, and locations of individual SNPs showing region-wide statistical significance in prior studies are indicated. The recombination rate in cM/Mb (right y-axis) is shown in orange. The horizontal grey dashed line represents the -log_10_ *p*-value for region-wide significance level (7.5 x 10^-5^).

In the NIMH multiplex collection, we performed TDT on 2209 SNPs in 186 nuclear families comprising of 408 trios. Although no SNP was significant after Bonferroni correction for multiple testing, one SNP in intron 1 showed marked association with ASD (rs17170073, *p* = 2.4 x 10^-4^) (Figure 1). The rs17170073 (MAF = 0.07) T allele is over-transmitted with the transmission frequency of τ = 0.69 (Table 1). We also analyzed the parent-of-origin effect of the transmission of rs17170073 alleles, demonstrating no significant transmission disequilibrium (τ_pat_ = 0.67, τ_mat_ = 0.72, *p* = 0.60). rs17170073 is in a large intron that has previously been reported to contain a microdeletion in an autistic patient [35]. The association of rs17170073 in the SSC was not significant (τ = 0.49, *p* = 0.91) (Table 1).

**Table 1:** Transmission disequilibrium of *CNTNAP2* variants identified in this study.

***SNP***	***MAF***	***STAGE***	***NIMH***					***SSC***					***NIMH + SSC***				
***(Minor/Major) allele***			***Trios***	***A1***	***A2***	***τ***	***P***	***Trios***	***A1***	***A2***	***τ***	***P***	***Trios***	***A1***	***A2***	***τ***	***P***
**rs17170073 (T/C)**	0.07	I	408	65	29	0.69	2 x 10^-4^	323	37	38	0.49	0.91	731	102	67	0.60	7.0 x 10^-3^
		II	1036	121	128	0.49	0.66										
		I + II	1445	186	157	0.54	0.12										
**rs2215798 (G/A)**	0.15	I	408	105	119	0.47	0.35	323	54	101	0.35	1.6 x10^-4^	731	159	220	0.42	1.7 x 10^-3^
		II						1479	351	351	0.50	1.0					
		I + II						1802	415	452	0.48	0.21					
**rs2710093 (G/C)**	0.17	I	408	85	132	0.39	1.4 x 10^-3^	323	67	108	0.38	1.9 x 10^-3^	731	152	240	0.39	9.0 x 10^-6^
		II	1089	273	287	0.49	0.55	1479	341	358	0.49	0.52	2568	614	645	0.49	0.38
		I + II	1497	358	419	0.46	2.8 x 10^-2^	1802	408	466	0.47	4.9 x 10^-2^	3299	766	885	0.46	3.4 x 10^-3^

Stage I samples were genotyped with TG-array and stage II samples were genotyped with either TaqMan or Sequenom assay. Trios: no of trios, A1: minor allele, A2: alternate allele, τ: transmission frequency of the minor allele, MAF: minor allele frequency, and P: p-value for τ

**Figure 2:**
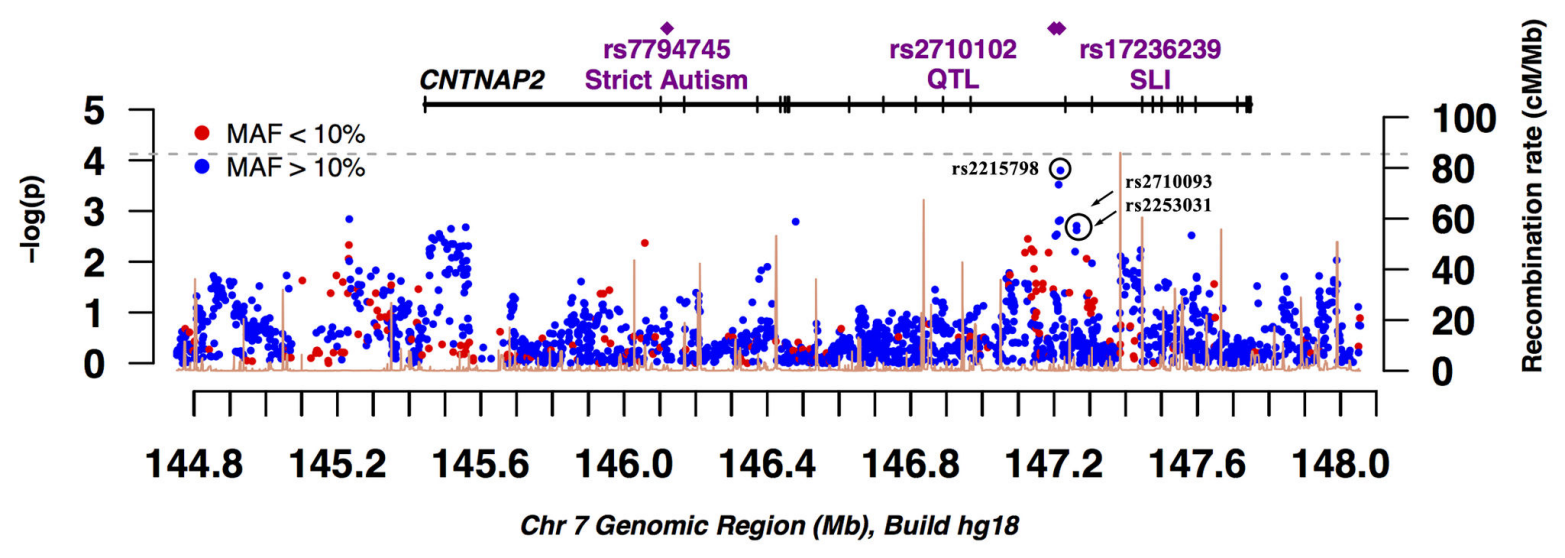
A plot of -log_10_ *p* across *CNTNAP2* from family-based association test (TDT) results on 323 simplex families.

In the SSC, following quality control, 2161 SNPs were analyzed in 323 parent-offspring trios. Like the TDT results in multiplex families, no SNP was significant after Bonferroni correction for multiple testing in the simplex families. However, two SNPs in intron 13 were marginally significant (rs2215798, *p* = 1.6 x 10^-4^; rs2708244, *p* = 3.0 x 10^-4^) (Figure 2) with an excess transmission from the father compared to the mother that was not statistically significant (rs2215798, τ_pat_ = 0.70, τ_mat_ = 0.60, *p* = 0.17; rs2708244, τ_pat_ = 0.70, τ_mat_ = 0.58, *p* = 0.12). rs2215798 and rs2708244 (MAF = 0.15) were highly correlated with each other (*r* ^2^ = 0.98). SNPs rs2215798 (τ = 0.47, *p* = 0.35) and rs2708244 (data not shown) had no significant genetic effect in the multiplex collection (Table 1). 

**Figure 3:**
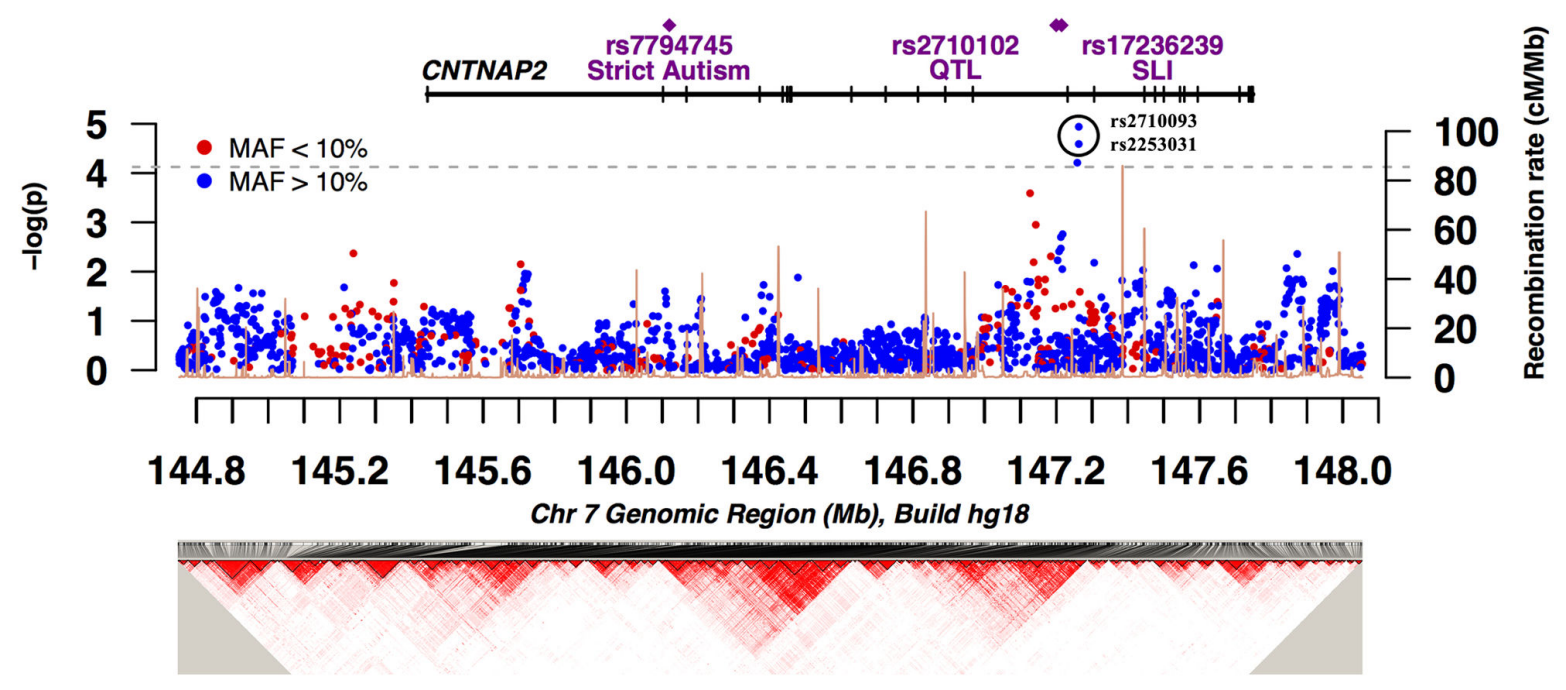
A plot of -log_10_ *p* across *CNTNAP2* from family-based association test (TDT) results on 509 merged multiplex and simplex families. The bottom panel shows a plot of all pairwise correlations (linkage disequilibrium, D’) between 2148 SNPs in this genomic interval.

TDT was also performed after merging the data from multiplex and simplex families. In the merged data (2148 SNPs in 731 affected offspring trios) two SNPs in intron 14 were significantly associated with autism (rs2710093; τ = 0.39, *p* = 9.0 x 10^-6^, rs2253031; τ = 0.39, *p* = 2.5 x 10^-5^), even after correcting for the number of SNPs tested by permutation (corrected empirical *p* = 0.01 and 0.03 respectively) (Figure 3 and Table 1). These two SNPs are highly correlated (*r* ^2^ = 0.99) and have a data completeness of 99.83% and 99.95%, one and zero observed Mendelian errors, and are in Hardy-Weinberg equilibrium (*p* = 0.14 and 0.25, respectively). Both SNPs have a minor allele frequency of 0.17. TDT based on the parent-of-origin of the allele did not indicate any transmission disequilibrium at rs2710093 (τ_pat_ = 0.65, τ_mat_ = 0.57, *p* = 0.10). A quantile-quantile (QQ) plot of the observed *p*-values revealed an overall good fit with the null distribution (Figure S2 in Information S1).

 In order to validate our finding from this study (rs17170073 in the multiplex, rs2215798 in the simplex and rs2710093 in merged dataset) we performed a stage II analysis by genotyping these three SNPs using TaqMan or Sequenom assays on an independent collection of 572 multiplex (1089 parent-offspring trios) and 1479 simplex (1479 parent-offspring trios) families. In addition, SNPs that were previously associated with strict autism and its endophenotypes (rs7794745, rs2710102 and rs17236239) were also genotyped (Table 2). The marginal genetic effects at rs17170073 (multiplex) and rs2215798 (simplex), and the significant genetic effect at rs2710093 seen in the merged dataset could not be validated in stage II samples (Table 1). Although no significant association was detected by TDT analysis at rs2710102 and rs17236239 genotyped in stage II in both the multiplex and simplex collections, rs7794745 showed marginal association (based on three SNP test) in the stage II multiplex collection (Table 2). This effect in the multiplex collection is in the same direction and similar to what we had previously reported [36]. We investigated if the lack of validation could be explained by diminished power to detect association (Supplementary Methods and Table S1 in Information S1). Table S1 indicates that for SNPs with significant findings in stage I (rs17170073, rs2215798 and rs2710093), there is considerable power in the additional families genotyped from both multiplex and simplex cohorts to detect the same size effect. For example, rs2710093 with GRR_Aa_ = 1.6 (240/152, Table 1) and allele frequency of 0.17, has >90% power in both the multiplex and simplex collections at an experiment-wise significance level of α = 7.5 x 10^-5^. Similarly, rs17170073 and rs2215798 with GRR_Aa_ = 2.2 (65/29, Table 1) and GRR_Aa_ = 1.8 (101/54, Table 1) respectively also have significant power to detect association in both collections. The failure to find such replicated effects suggests an incorrect hypothesis or heterogeneity.

**Table 2:** Transmission disequilibrium of *CNTNAP2* variants previously associated with ASD and autism related language traits.

***SNP***	***MAF***	***STAGE***	***NIMH***					***SSC***					***NIMH + SSC***				
***(Minor/Major) allele***			***Trios***	***A1***	***A2***	***τ***	***P***	***Trios***	***A1***	***A2***	***τ***	***P***	***Trios***	***A1***	***A2***	***τ***	***P***
**rs7794745 (T/A)**	0.39	I	408	197	185	0.52	0.54	323	146	136	0.52	0.55	731	343	321	0.52	0.3900
		II	1089	560	452	0.55	3.4 x 10^-4^	1479	654	619	0.51	0.17	2568	1214	1071	0.53	2.8 x 10^-3^
		I + II	1497	757	637	0.54	1.3 x 10^-3^	1802	800	755	0.51	0.25	3299	1557	1392	0.53	2.4 x 10^-3^
**rs2710102 (T/C)**	0.48	I	408	217	217	0.50	1.00	323	176	141	0.56	4.9 x 10^-2^	731	393	358	0.52	0.20
		II	1089	494	529	0.48	0.14	1479	690	672	0.51	0.32	2568	1184	1201	0.49	0.73
		I + II	1497	711	746	0.49	0.36	1802	866	813	0.52	0.19	3299	1577	1559	0.50	0.75
**rs17236239 (G/A)**	0.34	I	408	200	186	0.52	0.48	323	142	124	0.53	0.27	731	342	310	0.52	0.21
		II	1089	485	456	0.52	0.17	1479	578	591	0.49	0.35	2568	1063	1047	0.50	0.73
		I + II	1497	685	642	0.52	0.24	1802	720	715	0.50	0.89	3299	1405	1357	0.51	0.36

Stage I samples were genotyped with TG-array and stage II samples were genotyped with either TaqMan or Sequenom assay. SNPs rs7794745 and rs17236239 were genotyped only by TaqMan assay as these two SNPs were not in the TG-array panel. One-sided *p*-values are provided for stage II analysis of SNPs with prior association. Abbreviations are the same as in table 1.

We investigated the role of population substructure in the discordance of genetic effect between stage I and stage II samples. Using PCA analyses of genome-wide data available on the multiplex (stage I and II) and simplex (stage I) families we determined that the majority of the multiplex families (176/184 of the stage I and 360/501 stage II) and simplex families (243/317 of stage I) were of CEU ancestry (Figures S3A-C in Information S1). Consequently, TDT analyses were performed only in families with CEU ancestry (Table S2 in Information S1). These results indicate that in families with uniform ancestry, the strength of the genetic effect becomes stronger for rs7794745 in both multiplex (τ = 0.55 vs 0.54) and simplex (τ = 0.54 vs 0.51) collections; for rs17170073 in the multiplex (τ = 0.60 vs 0.54) collection only; and, for rs2215798 (τ = 0.35 vs 0.48), rs2710093 (τ = 0.40 vs 0.47) and rs2710102 (τ = 0.56 vs 0.52) in the simplex collection only. Although one cannot rule out the influence of random noise in effect size estimation due to changes in the sample size and composition, these associations did not reach region-wide significance and statistical power is an issue in the simplex collection (Table S1 in Information S1) given the smaller number of families analyzed. 

We also investigated the possibility that phenotypic differences between stage I and stage II families could explain the discordant results. A strict definition of diagnosis where an offspring was considered autistic if they scored positive for “autism” by both ADI-R and ADOS instruments was used. Although no SNP was significant after corrections for multiple testing, TDT analyses in strictly autistic individuals did strengthen the genetic effect at rs7794745 (τ= 0.57 vs 0.54) in the multiplex, and rs2710093 (τ = 0.37 vs 0.47) and rs2710102 (τ = 0.58 vs 0.52) in the simplex collection (Table S3 in Information S1). Since our analysis of phenotypic effects is grossly underpowered, the effect of random noise on effect size cannot be discounted (Table S1 in Information S1).

In order to evaluate association at untyped markers within this genomic interval we imputed genotypes at 809 and 3708 untyped SNPs using HapMap and 1000 Genomes as references. Following quality control (see Materials and Methods section), TDT analysis on imputed genotypes at untyped markers (455 SNPs from HapMap, 3167 SNPs from 1000 Genomes) did not reveal any additional association that was more significant than the ones we had already identified using genotyped markers, but revealed three additional SNPs with significant transmission disequilibrium (rs2710090; *p* = 9.5 x 10^-5^, rs2710091; *p* = 6.2 x 10^-5^, and rs1922888; *p* = 2.6 x 10^-5^). These three SNPs are in the same LD block and are highly correlated (*r* ^2^ = 0.96, 0.96, 0.99 respectively) with rs2710093 (Figure S4 in Information S1). This is not surprising given that our dense panel of SNPs afforded complete coverage of the genomic interval for variants with MAF >5%, and hence any existing association should be identified by our TG-array panel of SNPs and imputations only identified correlated SNPs with similar patterns of transmission disequilibrium.

We also performed a stratified TDT analysis by removing families with rare variants identified using published data on *CNTNAP2* gene and exome sequencing (see Supplementary Methods and Figures S5A and S5B in Information S1). Although no SNP remained significant after Bonferroni correction, one SNP reached marginal statistical significance in both the multiplex (rs10260544, *p* = 1.1 x 10^-4^, MAF = 6%, Missing = 3.7%, and no Mendelian errors) and simplex collection (rs10488072, *p* = 6.4 x 10^-4^, MAF = 7.5%, Missing = 0.3%, and no Mendelian errors). 

 Given the dense panel of SNPs in the custom designed TG-array, it should be possible to use raw intensity information from each SNP for copy number analysis. Our attempts in this exercise proved unsuccessful, owing primarily to the fact that the custom array was designed as a genotyping assay for allelic discrimination, frequently leading to signal saturation and significant variation in intensity between arrays. This prevented us from resolving copy number states accurately. However, we used Mendelian segregation errors to look for deletion polymorphisms in each family. Given a set of genotypes for parents and offspring it is possible to identify deletions and its parental origin by evaluating the presence of contiguous Mendelian errors. Using this method, we did not identify any deletions except in one family in whom the deletion has been previously reported [35].

### CNTNAP2 expression is altered in autism-affected cortex

 We next assembled a cortical sample series from 39 autism cases and 44 controls from the Autism Tissue Program and the NICHD Brain and Tissue Bank for Developmental Disorders. The series is matched for region (Brodmann area 19 of the occipital cortex), age (mean 22.8±2.9 years cases and 21.1±2.2 years controls, median age 18 and 17 years, case/control respectively), sex (74% male in cases and 75% males in controls) and post-mortem interval (20.9 ± 1.6 hours cases and 17.5±1.0 hours controls, median 21 and 18 hours cases/controls respectively). The BA19 somatosensory/visual association region was selected because the occipital cortex is highly comparable on a gross structural level between autism and control [55], reducing the potential effect of gross tissue heterogeneity on expression levels, and because defects in connectivity have been implicated in this area [56-61]. 

**Figure 4:**
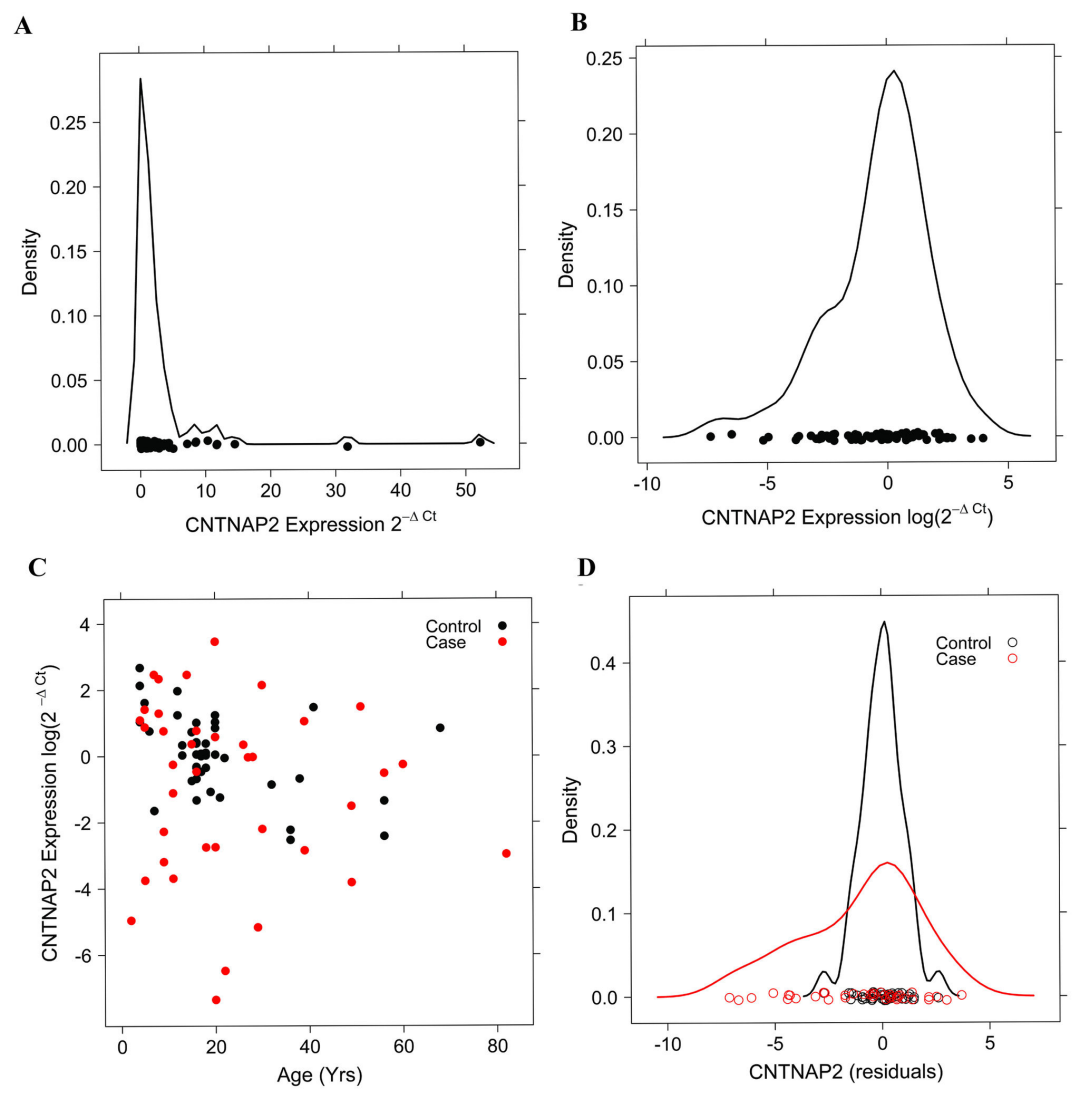
*CNTNAP2* expression in autism and control brains. (A) *CNTNAP2* expression normalized to *TBP* and (B) log transformed. (C) *CNTNAP2* expression as a function of age in cases (red dots) and controls (black dots), and (D) *CNTNAP2* expression in autism and control brains adjusted for age and age^2^ effects.

 To determine whether changes in *CNTNAP2* expression levels occur in the cortex in autism, we developed a quantitative RT-PCR assay with amplification conditions optimized to allow efficiencies of 1.0 ± 0.05. cDNA library integrity was functionally verified with probe sets that target the 5’ or 3’end of low-abundance mRNA transcripts. Relative expression was determined using the *TBP* probe (Figure 4A) and the association of log transformed expression levels (Figure 4B) with potential confounders (age, age ^2^, Sex, PMI, site of collection) was determined in controls using linear regression models. Only age and age^2^ were significant, with decreased expression associated with increasing age (Figure 4C). Residuals were calculated using β estimates derived from the controls (under the assumption that expression levels in cases are likely to be altered). Using a logistic expression analysis we observed that altered *CNTNAP2* expression is highly associated with autism status (*p* = 1.9 x10^-5^), with both high and low expression observed in cases, though low levels were more prevalent (Figure 4D). We also assessed normalization to other housekeeping genes including *GADPH* and neuron-specific *MAP2* with similar results. 

## Discussion

The existing view of the genetic architecture of autism is that numerous genes are involved, but we do not have enough evidence to distinguish between simple heterogeneity (many genes are involved but each patient harbors mutations at only one gene) and multigenic inheritance (each patient harbors mutations at multiple genes). There are two reasons for this deficiency. First, it is generally believed that different types of sequence variants impart quantitatively different genetic risks and are, therefore, enriched in different types of families: for example, multiplex families are enriched for rare variants of large effect [62,63] whereas simplex families harbor common variants of small effect [64] or *de novo* mutations [65-67]. Second, there are no comprehensive genome-wide studies of different susceptibility variants (rare/common, SNPs/CNVs, segregating/*de novo*) in a common but a large set of families to enhance gene discovery and assess the relative weight of each variant type. This study was to correct this second deficiency using *CNTNAP2* as an exemplar, since existing studies already suggested that rare deleterious mutations [34], complex chromosomal alterations [34,38,39], and common variants [35-37] of minor genetic effect all predispose to ASD and overlapping [40-43,68,69] neuropsychiatric conditions.

In this study, we evaluated the broader role of common variants in 731 multiplex and simplex families by performing fine-mapping of the *CNTNAP2* gene locus at high density using a custom genotyping panel. Our laboratory has reported an association at rs7794745 for ASD, with a significant over-transmission from the mother compared to the father [36]. Our current study, despite genotyping rs7794745 in a larger number of multiplex trios, failed to show a region-wide significant association, although nominal association and transmission disequilibrium that gets stronger after correcting for ancestry and strict autism diagnosis persists. Nevertheless, we demonstrate moderately significant association at rs2710093 in both multiplex and simplex families that survived multiple test corrections in the combined data. However, this genetic effect at rs2710093 could not be validated in an independent collection of 2568 trios. SNPs that were previously associated with autism endophenotypes were not associated with ASD in the current study. In addition, we performed a stratified TDT analyses by removing families based on published *CNTNAP2* sequence variants suspected to be mutations. No SNP reached significance in 109 multiplex and 116 simplex families without *CNTNAP2* rare variants.

One should question why despite dense common variant coverage at *CNTNAP2* we did not identify any association signal that withstood both multiple-testing correction and independent validation. While power may have been an issue in the some stratified analyses, including analyses in families with strict definition of autism and in families with absence of rare variants, it is not the case with the stage II validation cohort. This is particularly intriguing given our gene expression results. It is possible that the original finding represents a false positive finding due to population substructure or diagnostic differences between stage I and stage II samples. However, we found no evidence for either in the non-replication of the original finding. It is likely that biological endophenotypes such as brain size, IQ and prenatal history or behavioral endophenotypes such as language, social interaction and face processing could modify disease association. In fact, it has been known for some time now that the concordance rates of these broader spectrum of phenotypic traits among monozygotic and dizygotic twins is higher than that for the categorical/behavioral diagnosis of “autism”, signifying a greater genetic liability to these endophenotypes [5-7]. Consequently, it is likely that inconsistent replication in independent samples could reflect failure to take various endophenotypes that are indicators of genetic heterogeneity into account [70]. This is a general problem in autism and not unique to this study. If true, this implies that autism genetic studies will be frustrating until we account for significant endophenotypes.

A simpler explanation may suffice. It is likely that *CNTNAP2* is involved in autism. Numerous genetic, neurobiological, imaging and mouse model studies have amply clarified that *CNTNAP2* plays a crucial role in ASD and other related neurodevelopmental disorders. This study simply shows that its overall burden in ASD cannot be large. We present evidence for the limited role of common variants at *CNTNAP2* for ASD in both multiplex and simplex families. With autism being a spectrum disorder with broad range of symptoms and severities, a further detailed analysis of endophenotypes will shed light on the gene-phenotype relationships within each homogeneous subtype, with each subtype having potentially different genetic liabilities.

To further evaluate the role of *CNTNAP2* in autism, we performed the first gene expression study in post-mortem brain samples of autistic individuals. In mice, *CNTNAP2*’s highest expression is in the cerebral cortex with ubiquitous distribution across the cortical mantle (Allen Brain Atlas). We assembled a large series of brain samples from the cortex of autism and controls. In corroboration with the general lack of detection of deletions or other CNVs in autism families, all autism brain samples demonstrated *CNTNAP2* expression despite the presumptive unstable locus (FRA7I) spanning the gene. However, *CNTNAP2* demonstrated both significantly low- and high-expression in autism cases compared to controls (*p* = 1.9 x10^-5^). Whether this altered expression is caused by cis-acting or trans-acting factors in the *CNTNAP2* locus was not resolved in this study, nor whether low or high expression might be deleterious, advantageous, or benign, with respect to the autism phenotype. Complete loss of *CNTNAP2* expression in mice causes profound neurodevelopmental problems that at least include altered sorting of potassium channel in myelinated neurons. The effects of over-expression of *CNTNAP2* have not been evaluated. Our studies provide an intriguing initial insight into *CNTNAP2* in that the suggested linkage of the gene to autism likely extends beyond simple haploinsufficiency. 

### Web Resources

The URLs for data presented herein are as follows:

plink, http://pngu.mgh.harvard.edu/~purcell/plink/
Haploview, http://www.broad.mit.edu/mpg/haploview
Online Mendelian Inheritance in Man (OMIM), http://www.ncbi.nlm.nih.gov/Omim
Hapmap, http://hapmap.ncbi.nlm.nih.gov/
1000 Genomes Project, http://www.1000genomes.org/
MaCH, http://www.sph.umich.edu/csg/yli/mach/index.html
BEAGLE, http://faculty.washington.edu/browning/beagle/beagle.html

## Supporting Information

Information S1 **Combined Supplementary Methods, Supplementary Tables and Supplementary Figures file.** Additional details of the methods are provided in Supplementary Methods section. Supplemental tables and figures are provided in Supplementary Tables and Supplementary Figures section.
(DOCX)
