# Correction: Defining the Contribution of *CNTNAP2* to Autism Susceptibility

**DOI:** 10.1371/annotation/b4552fc7-285e-42e8-b126-d498eaf9f73a

**Published:** 2013-12-20

**Authors:** Srirangan Sampath, Shambu Bhat, Simone Gupta, Ashley O’Connor, Andrew B. West, Dan E. Arking, Aravinda Chakravarti

Author Maria X. Sosa is missing from the author byline. Dr. Sosa should be listed as the fifth author and affiliated with institution number 1, Center for Complex Disease Genomics, McKusick-Nathans Institute of Genetics Medicine, Johns Hopkins University School of Medicine, Baltimore, Maryland, United States of America. Sosa should be noted as having made the following contributions to the article: Performed the experiments. The Citation should read: Sampath S, Bhat S, Gupta S, O’Connor A, Sosa MX, et al. (2013) Defining the Contribution of CNTNAP2 to Autism Susceptibility. PLoS ONE 8(10): e77906. doi:10.1371/journal.pone.0077906.

In addition, the first author, Srirangan Sampath, was incorrectly indicated as being the Corresponding Author of the article. The correct Corresponding Author is the last author, Aravinda Chakravarti. The last author's email address is already correct as indicated for the first author.

In addition, the last author was incorrectly indicated as having changed affiliation to the institution now indicated as their Current Address, Department of Molecular and Human Genetics, Baylor College of Medicine, Houston, Texas, United States of America. This institution is the Current Address of the first author, not the last author. 

