# Supplementary material for: Defining the Contribution of CNTNAP2 to Autism Susceptibility
Source: PLoS One. 2013 Oct 17;8(10):e77906. doi: 10.1371/journal.pone.0077906 (PMC3798378; doi:10.1371/journal.pone.0077906)

**Supporting Information S1**

Defining the contribution of *CNTNAP2* to autism susceptibility

Srirangan Sampath^1,*^, Shambu Bhat^1^, Simone Gupta^1^, Ashley O’Connor^1^, Andrew B. West^2^, Dan E. Arking^1^, and Aravinda Chakravarti^1§^.

^1^Center for Complex Disease Genomics, McKusick-Nathans Institute of Genetic Medicine, Johns Hopkins University School of Medicine, Baltimore, MD, USA.

^2^Department of Neurology and Neurobiology, Center for Neurodegeneration and Experimental Therapeutics, University of Alabama at Birmingham, Birmingham, Alabama, USA

^*^ Current address, Department of Molecular and Human Genetics, Baylor College of Medicine, Houston, TX, USA

**Table of Contents**

**SUPPLEMENTARY METHODS**

**TG-array Genotyping assay………………………………………………2**

**Custom TaqMan assay primer sequences for rs2710093………………..3**

**Power analysis……………………………………………………………..3**

**Correction for multiple testing……………………………………………4**

**Imputation analysis………………………………………………………..5**

**Stratified TDT analysis……………………………………………………6**

**PLINK vs FBAT…………………………………………………………...7**

**References………………………………………………………………….8**

**SUPPLEMENTARY TABLES**

**Table S1…………………………………………………………………….10**

**Table S2…………………………………………………………………….11**

**Table S3…………………………………………………………………….12**

**SUPPLEMENTARY FIGURES**

**Figure Legends…………………………………………………………….13**

**Figures S1A & S1B………………………………………………………...15**

**Figure S2…………………………………………………………………...16**

**Figure S3A…………………………………………………………………17**

**Figure S3B…………………………………………………………………18**

**Figure S3C…………………………………………………………………19**

**Figure S4…………………………………………………………………...20**

**Figure S5A…………………………………………………………………21**

**Figure S5B…………………………………………………………………22**

**SUPPLEMENTARY METHODS**

**TG-array Genotyping assay**

Both multiplex and simplex samples were genotyped using TG-arrays according to the manufacturer’s protocol. Briefly, two micrograms of genomic DNA were hybridized with Molecular Inversion probes (MIP) overnight at 58°C. The following day, gaps in the MIPs were filled with dNTPs in four separate reactions, and were ligated to form circular padlock probes. This was followed by exonuclease digestion to remove linear MIPs and single-stranded DNA molecules, leaving behind only undigested circular padlock probes. The circular probes were linearized by cleaving prior to PCR amplification using common primers. A second set of allele-specific primers were used to amplify and label each product. The labeled PCR products were then cleaved to release the MIP tag and an allele-specific label. The four separate allele-specific labeled MIP for each sample were then pooled and hybridized to TG-arrays at 39°C for 16 hr. The arrays were stained and washed on a GeneChip^®^ Fluidics Station 450 (Affymetrix) and scanned on a GeneChip^®^ Scanner 3000 7G 4C (Affymetrix). The genotypes were called using Affymetrix GeneChip Targeted Genotyping Analysis Software (GTGS) (v1.6) [1].

**Custom TaqMan assay primer sequences for rs2710093**

Forward Primer: AGATGAGTACAGAGACTTTTTGAATAACTAATTTAATTAATGA

Reverse Primer: AAAACAAAAAACTTTGCATTAGATTACTTTTCAAT

Reporter 1 (VIC): CTGTATGAGCAAAGGAC

Reporter 2 (FAM): CTGTATGACCAAAGGAC

**Power analysis**

Power analysis was performed using the Genetics Power Calculator tool available at pngu.mgh.harvard.edu/~purcell/gpc. Table S1 indicates the number of parent-child trios needed to attain 90% power to detect association at different significance thresholds of α = 0.05 (point-wise significance), α = 7.5 x 10^-5^ (experiment-wise significance), and α = 5 x 10^-8^ (genome-wise significance), multiplicative and additive modes of inheritance, and a range of genotype relative risk (GRR_Aa_) and disease allele frequency (pA). In all cases we assumed the following: complete linkage disequilibrium between the trait and the marker locus (i.e., D′ = 1, a fair assumption given the high coverage provided by our custom array density), the marker and the disease loci have equal allele frequencies, and a disease prevalence of 0.01 (although disease prevalence does not affect power of a TDT for a given value of GRR and mode of inheritance). It is clear from this table that for SNPs with significant findings in stage I (rs17170073, rs2215798 and rs2710093) we have considerable power to detect association in the stage II validation cohort. And, for SNPs with prior evidence of association (rs7794745, rs2710102 and rs17236239), there is >90% power only in an additive model of inheritance, and significant loss of power in a multiplicative model of inheritance in stage II. There is loss of power in some of the sub-analyses, especially in the analysis of families with strict diagnosis of autism, and in families with absence of rare variants.

**Correction for multiple testing**

In order to have an assessment of the effective number of SNPs for multiple testing correction we used an independent method to judge statistical significance that is based on principle component analysis (PCA). This method relies on calculating the effective number of SNPs (M_eff_) for multiple testing in association studies [2]. M_eff_, as estimated by Nyholt (2004) uses the SNPSpD software, is known to be conservative (overestimates M_eff_) in the presence of significant correlation between SNPs. Hence we used M_eff-Li_ as an estimate of the effective no of SNPs based on the method proposed by Li and Ji (2005) [3]. In the combined dataset of multiplex and simplex families (2148 SNPs in 731 affected-offspring trios), 61 redundant SNPs in complete LD (*r*^2^ = 1) with another SNP were removed before analysis using SNPSpD. Based on this modified method, we estimated M_eff-Li_ to be 663.12, giving an experiment-wise significance threshold required to keep the overall type-I error rate of 5% as 7.5 x 10^-5^ (0.05/663.12).

**Imputation analysis**

Imputations were performed using two methods, the Markov Chain Haplotyping software [4] (MaCH v1.0) and BEAGLE (v3.3.0) [5], using phased data from HapMap Phase III [6] (http://hapmap.ncbi.nlm.nih.gov/downloads/phasing/2009-02_phaseIII/HapMap3_r2/) and the March 2010 release (pilot 1) of 1000 Genomes [7] (ftp://ftp.1000genomes.ebi.ac.uk/vol1/ftp/pilot_data/release/2010_03/pilot1/). MaCH was run with default options using a 2-step imputation process, first by estimating the model parameters, then using the estimated parameters to impute genotypes at untyped markers for all samples. To estimate the model parameters that relate the genotyped samples to the reference haplotypes, each dataset was run with 100 iterations (“--rounds 100” option) using a representative subset of 350 unrelated individuals from this study. In step-2, each sample was imputed independently using the model parameters estimated from step-1 (“--greedy --mle --mldetails –autoFlip” option). For BEAGLE analysis, the 731 trios (408 multiplex and 323 simplex) were first phased using the reference haplotypes before imputing genotypes at untyped SNPs. BEAGLE uses Mendelian constraints for phasing parent-offspring trios, with multiple affected sibs from each family imputed as independent parent-offspring trios. Although this violates BEAGLE’s assumption of independent trios, the large number of families analyzed here is expected to produce unbiased results [8]. For imputation with HapMap Phase III data, phased haplotypes (N=1084) from CEU, YRI, TSI, MEX, CHB and JPT populations were used as reference. 1718 HapMap SNPs overlapped with the TG-array SNPs, of which 909 were common to both HapMap and the TG-array, and 1239 SNPs were unique to the TG-array. All samples in this study were then imputed for 809 untyped HapMap SNPs, and subsequently filtered to retain only high-quality SNPs (643 SNPs with high quality, *r^2^*_MACH_ >0.80). Following SNP quality control (inclusion criteria mentioned under statistical analysis section), the TDT analysis was performed on 455 imputed SNPs. Imputation was also performed using the 362 phased haplotypes (120 CEU, 118 YRI, 124 CHB & JPT populations) from the 1000 Genomes pilot1. Of the 5395 SNPs from this resource that overlapped with the TG-array, 1687 were common to both HapMap and the TG-array, and 461 were unique to the TG-array. For all samples, 3708 untyped 1000 Genomes SNPs were imputed. Following SNP quality control, TDT analysis was performed on 3167 imputed SNPs.

**Stratified TDT analysis**

Among the families we had genotyped in this study, we identified multiplex and simplex families that do not carry a *CNTNAP2* rare variants based on published data. Given the hypothesis that rare sequence variants (highly penetrant rare variants of large effect in the multiplex and *de novo* deleterious variants in the simplex) impart quantitatively different genetic liability that is independent from common variants, we preformed a stratified analysis by removing families carrying *CNTNAP2* rare variants before performing family based association analysis. First we identified all overlaps between samples that were genotyped with TG-array and published *CNTNAP2* gene sequencing [9] or exome data [10-12]. Of the 186 multiplex families (408 trios) genotyped with TG-array, 110 families overlapped with Bakkaloglu *et al*, of which 109 families (238 trios) were classified as not having rare variants. Whereas, none of the 744 simplex families that were sequenced carried *de novo* *CNTNAP2* variants [10-12]. Of the 323 simplex families (323 trios) genotyped with TG-array, 116 families overlapped with the three exome sequencing studies and were all classified as not carrying a *de novo* *CNTNAP2* variant. After quality control (see Methods), we performed TDT analysis on more than 2100 SNPs in 109 multiplex and 116 simplex families. Although no SNP remained significant after Bonferroni correction one SNP reached marginal statistical significance in both the multiplex (rs10260544, *p* = 1.1 x 10^-4^, MAF = 6%, Missing = 3.7%, and no Mendelian errors) and simplex collection (rs10488072, *p* = 6.4 x 10^-4^, MAF = 7.5%, Missing = 0.3%, and no Mendelian errors).

**PLINK vs FBAT**

We performed family based association analysis on 731 combined multiplex and simplex affected offspring trios using both PLINK and FBAT to test for the effect of genotype correlation in multiplex families. The following *p*-values demonstrate that this bias is small.

| **SNP** | **PLINK**  **(*p* values)** | **FBAT**  **(*p* values)** |
| --- | --- | --- |
| **rs17170073** | 7.0 x 10^-3^ | 7.4 x 10^-3^ |
| **rs2215798** | 1.7 x 10^-3^ | 1.8 x 10^-3^ |
| **rs2710093** | 1.1 x 10^-5^ | 2.6 x 10^-5^ |

**References**

1. Moorhead M, Hardenbol P, Siddiqui F, Falkowski M, Bruckner C, et al. (2006) Optimal genotype determination in highly multiplexed SNP data. Eur J Hum Genet 14: 207-215.
2. Nyholt DR (2004) A simple correction for multiple testing for single-nucleotide polymorphisms in linkage disequilibrium with each other. Am J Hum Genet 74: 765-769.
3. Li J, Ji L (2005) Adjusting multiple testing in multilocus analyses using the eigenvalues of a correlation matrix. Heredity (Edinb) 95: 221-227.
4. Li Y, Willer CJ, Ding J, Scheet P, Abecasis GR (2010) MaCH: using sequence and genotype data to estimate haplotypes and unobserved genotypes. Genet Epidemiol 34: 816-834.
5. Browning BL, Browning SR (2009) A unified approach to genotype imputation and haplotype-phase inference for large data sets of trios and unrelated individuals. Am J Hum Genet 84: 210-223.
6. The International HapMap Consortium (2007). A second generation human haplotype map of over 3.1 million SNPs. Nature 449: 851-861.
7. Durbin RM, Abecasis GR, Altshuler DL, Auton A, Brooks LD, et al. (2010) A map of human genome variation from population-scale sequencing. Nature 467: 1061-1073.
8. Nelson S, Laurie CC, Crosslin D, Browning BL, Marazita ML Effects of ignoring relatedness among study subjects in genotype imputation analyses. 60th Annual Meeting of the American Society of Human Genetics, 2-6 November 2010. Washington, DC (abstract 3030).
9. Bakkaloglu B, O'Roak BJ, Louvi A, Gupta AR, Abelson JF, et al. (2008) Molecular cytogenetic analysis and resequencing of contactin associated protein-like 2 in autism spectrum disorders. Am J Hum Genet 82: 165-173.
10. Iossifov I, Ronemus M, Levy D, Wang Z, Hakker I, et al. (2012) De novo gene disruptions in children on the autistic spectrum. Neuron 74: 285-299.
11. O'Roak BJ, Vives L, Girirajan S, Karakoc E, Krumm N, et al. (2012) Sporadic autism exomes reveal a highly interconnected protein network of de novo mutations. Nature 485: 246-250.
12. Sanders SJ, Murtha MT, Gupta AR, Murdoch JD, Raubeson MJ, et al. (2012) De novo mutations revealed by whole-exome sequencing are strongly associated with autism. Nature 485: 237-241.

**SUPPLEMENTARY TABLES**

|  |  |  | **α = 0.05** | | **α = 7.5 x 10^-5^** | | **α = 5.0 x 10^-8^** | |
| --- | --- | --- | --- | --- | --- | --- | --- | --- |
| **SNP** | **GRR_Aa_** | **pA** | **Multiplicative** | **Additive** | **Multiplicative** | **Additive** | **Multiplicative** | **Additive** |
| **rs7794745** | **1.06** | 0.39 | 1.3 x10^4^ | 327 | 3.5 x 10^4^ | 854 | 5.8 x 10^4^ | 1408 |
| **rs2710102** | **1.09** | 0.48 | 6144 | 227 | 1.6 x10^4^ | 593 | 2.6 x10^4^ | 978 |
| **rs17236239** | **1.10** | 0.34 | 5084 | 359 | 1.3 x10^4^ | 939 | 2.1 x10^4^ | 1549 |
| **rs2710093** | **1.60** | 0.17 | 297 | 231 | 775 | 602 | 1278 | 994 |
| **rs2215798** | **1.80** | 0.15 | 202 | 184 | 528 | 481 | 872 | 794 |
| **rs17170073** | **2.20** | 0.07 | 195 | 202 | 509 | 528 | 839 | 871 |

**Table S1:** Number of parent-child trios needed to obtain 90% power to detect association at effect sizes observed for SNPs in stage I analysis. The number of trios were calculated for various levels of significance (α). Assumptions include complete linkage disequilibrium between the trait and the marker locus A, equal allele frequencies at trait and marker loci, and multiplicative and additive modes of inheritances. GRR_Aa_: Genotype relative risk for Aa, pA: marker allele frequency.

| **SNP** | **MAF** | **NIMH** | | | | | **SSC** | | | | | **NIMH + SSC** | | | | |
| --- | --- | --- | --- | --- | --- | --- | --- | --- | --- | --- | --- | --- | --- | --- | --- | --- |
| ***(Minor/Major) allele*** |  | ***Trios*** | ***A1*** | ***A2*** | ***τ*** | ***P*** | ***Trios*** | ***A1*** | ***A2*** | ***τ*** | ***P*** | ***Trios*** | ***A1*** | ***A2*** | ***τ*** | ***P*** |
| **rs17170073 (T/C)** | 0.07 | 1006 | 148 | 98 | 0.60 | 1.4 x 10^-3^ | 243 | 31 | 29 | 0.52 | 0.80 | 1249 | 179 | 127 | 0.58 | 2.9 x 10^-3^ |
| **rs2251798 (G/A)** | 0.15 | 383 | 101 | 112 | 0.47 | 0.45 | 243 | 39 | 74 | 0.35 | 9.9 x 10^-4^ | 626 | 140 | 186 | 0.43 | 1.1 x 10^-2^ |
| **rs2710093 (G/C)** | 0.17 | 1041 | 227 | 265 | 0.46 | 8.6 x 10^-2^ | 243 | 48 | 73 | 0.40 | 2.3 x 10^-2^ | 1284 | 275 | 338 | 0.45 | 1.1 x 10^-2^ |
| **rs7794745 (T/A)** | 0.39 | 1041 | 550 | 445 | 0.55 | 8.7 x 10^-4^ | 243 | 115 | 97 | 0.54 | 0.22 | 1284 | 665 | 542 | 0.55 | 4.0 x 10^-4^ |
| **rs2710102 (T/C)** | 0.48 | 1041 | 518 | 528 | 0.50 | 0.76 | 243 | 128 | 101 | 0.56 | 7.4 x 10^-2^ | 1284 | 646 | 629 | 0.51 | 0.63 |
| **rs17236239 (G/A)** | 0.34 | 1041 | 493 | 473 | 0.51 | 0.52 | 243 | 103 | 94 | 0.52 | 0.52 | 1284 | 596 | 567 | 0.51 | 0.40 |

**Table S2:** TDT analysis in families of European ancestry. The specific ancestry in multiplex and simplex families was determined using existing genome-wide data and principal component analysis. As shown from the table, population stratification did not explain the absence of association; abbreviations are the same as in table 1.

| **SNP** | **MAF** | **NIMH** | | | | | **SSC** | | | | | **NIMH + SSC** | | | | |
| --- | --- | --- | --- | --- | --- | --- | --- | --- | --- | --- | --- | --- | --- | --- | --- | --- |
| ***(Minor/Major) allele*** |  | ***Trios*** | ***A1*** | ***A2*** | ***τ*** | ***P*** | ***Trios*** | ***A1*** | ***A2*** | ***τ*** | ***P*** | ***Trios*** | ***A1*** | ***A2*** | ***τ*** | ***P*** |
| **rs17170073 (T/C)** | 0.07 | 567 | 62 | 62 | 0.50 | 1.00 | 240 | 28 | 27 | 0.51 | 0.89 | 807 | 90 | 89 | 0.50 | 0.94 |
| **rs2251798 (G/A)** | 0.15 | 137 | 41 | 47 | 0.47 | 0.52 | 240 | 40 | 73 | 0.35 | 1.9 x 10^-3^ | 377 | 81 | 120 | 0.40 | 6.0 x 10^-3^ |
| **rs2710093 (G/C)** | 0.17 | 580 | 135 | 161 | 0.46 | 0.13 | 240 | 49 | 84 | 0.37 | 2.4 x 10^-3^ | 820 | 184 | 245 | 0.43 | 3.2 x 10^-3^ |
| **rs7794745 (T/A)** | 0.39 | 580 | 322 | 246 | 0.57 | 1.4 x 10^-3^ | 307 | 132 | 138 | 0.49 | 0.72 | 887 | 454 | 384 | 0.54 | 1.6 x 10^-2^ |
| **rs2710102 (T/C)** | 0.48 | 580 | 278 | 278 | 0.50 | 1.00 | 240 | 138 | 101 | 0.58 | 1.7 x 10^-2^ | 820 | 416 | 379 | 0.52 | 0.19 |
| **rs17236239 (G/A)** | 0.34 | 580 | 270 | 267 | 0.50 | 0.90 | 307 | 119 | 122 | 0.49 | 0.85 | 887 | 389 | 389 | 0.50 | 1.00 |

**Table S3:** TDT analysis in trios with a strict diagnosis of autism (positive for both ADI-R and ADOS instruments); abbreviations are the same as in table 1.

**SUPPLEMENTARY FIGURES**

**Supplementary Figure Legends**

**Figure S1:** Minor allele frequency (MAF) of TG-array SNPs. (A) A plot of MAF of TG-array SNPs across the *CNTNAP2* locus. A horizontal line represents the *CNTNAP2* gene with vertical hash marks indicating exons. The recombination rate in cM/Mb (right y-axis) is shown in orange. (B) A histogram of MAF demonstrating that the majority of SNPs have frequency >5%.

**Figure S2:** Q-Q Plot. Quantile-quantile plot comparing observed and expected -log_10_-transformed *p*-values for TDT on the 731 combined multiplex and simplex trios. The observed p-values at the extreme right are consistent with a level of evidence for association that departs from the random expectation.

**Figure S3:** The top two axes of variation from principal components analysis (PCA) are plotted for HapMap II and ASD samples from (A) 317 Stage I simplex samples (SSC), (B) 184 Stage I multiplex samples, and (C) 501 Stage II multiplex samples. The top two axes contribute to 11% of the total variance and are plotted for HapMap reference populations of CEU (90), YRI (90), CHB (45) and JPT (45). The majority of the samples we examined are of European ancestry with the inset focusing on the CEU cluster.

**Figure S4:** Linkage disequilibrium patterns (pairwise correlations, *r*^2^) of genotyped and imputed SNPs. Pairwise correlations of 26 SNPs in intron 14 around rs2710093 are shown. Values within each diamond represents the *r*^2^ values; entries without values have *r*^2^ = 1.0. Solid lines bound LD blocks, the shading representing the strength of correlation between SNPs, with white to shades-of-grey to black reflecting lower to higher values of *r*^2^.

**Figure S5:** Family-based association test (TDT) results from (A) 109 multiplex, and (B) 116 simplex families. The physical position within the *CNTNAP2* locus in megabases (Mb) and the -log_10_ *p*-values are shown on the x and y-axes, respectively. The SNPs with MAF <10% and those with MAF ≥10% are shown as red and blue filled circles, respectively. The *CNTNAP2* gene is represented by a horizontal line, with vertical hash marks indicating exons, and locations of individual SNPs showing genome-wide statistical significance in prior studies indicated. The recombination rate in cM/Mb (right y-axis) is shown in orange.

**Figure S1**

**A**


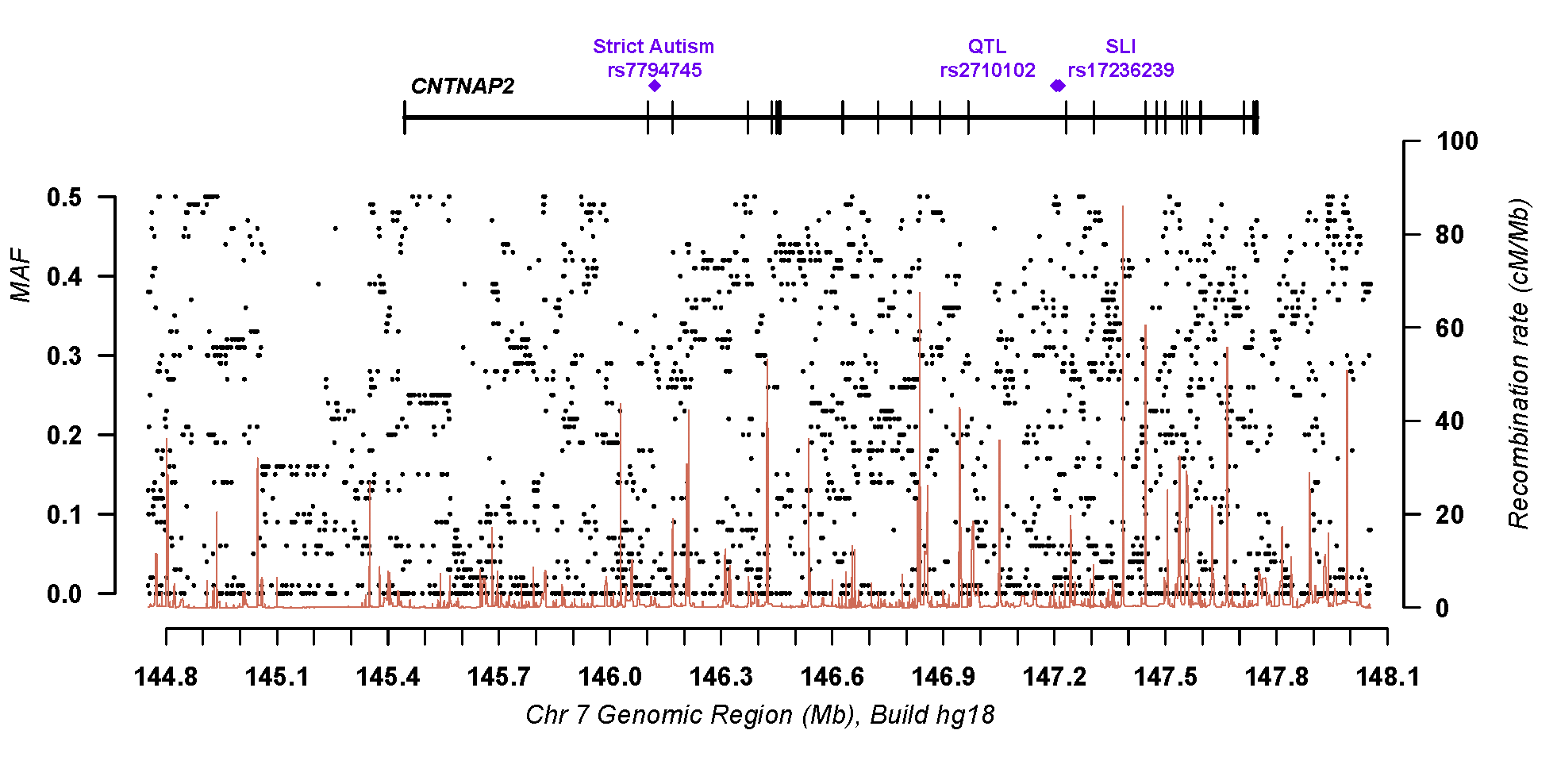


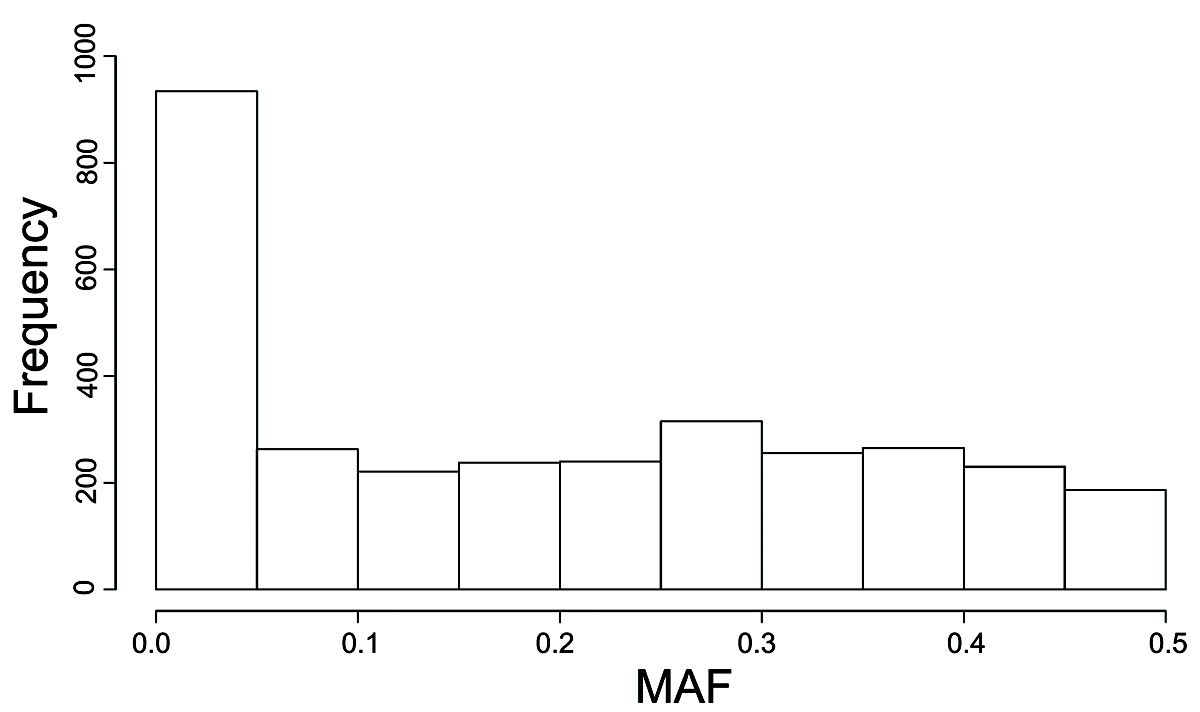
B

**Figure S2**

**
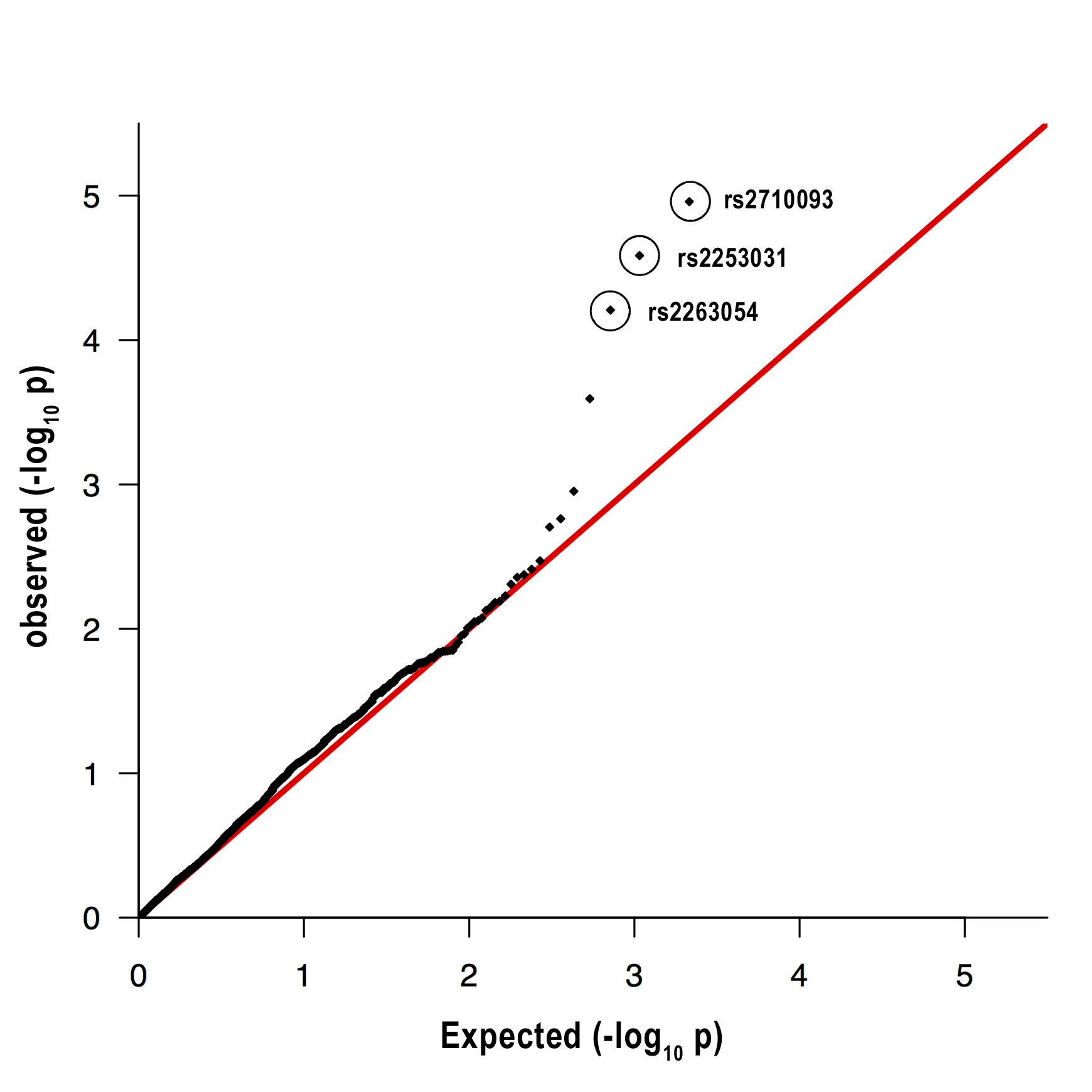
**

**Figure S3A**


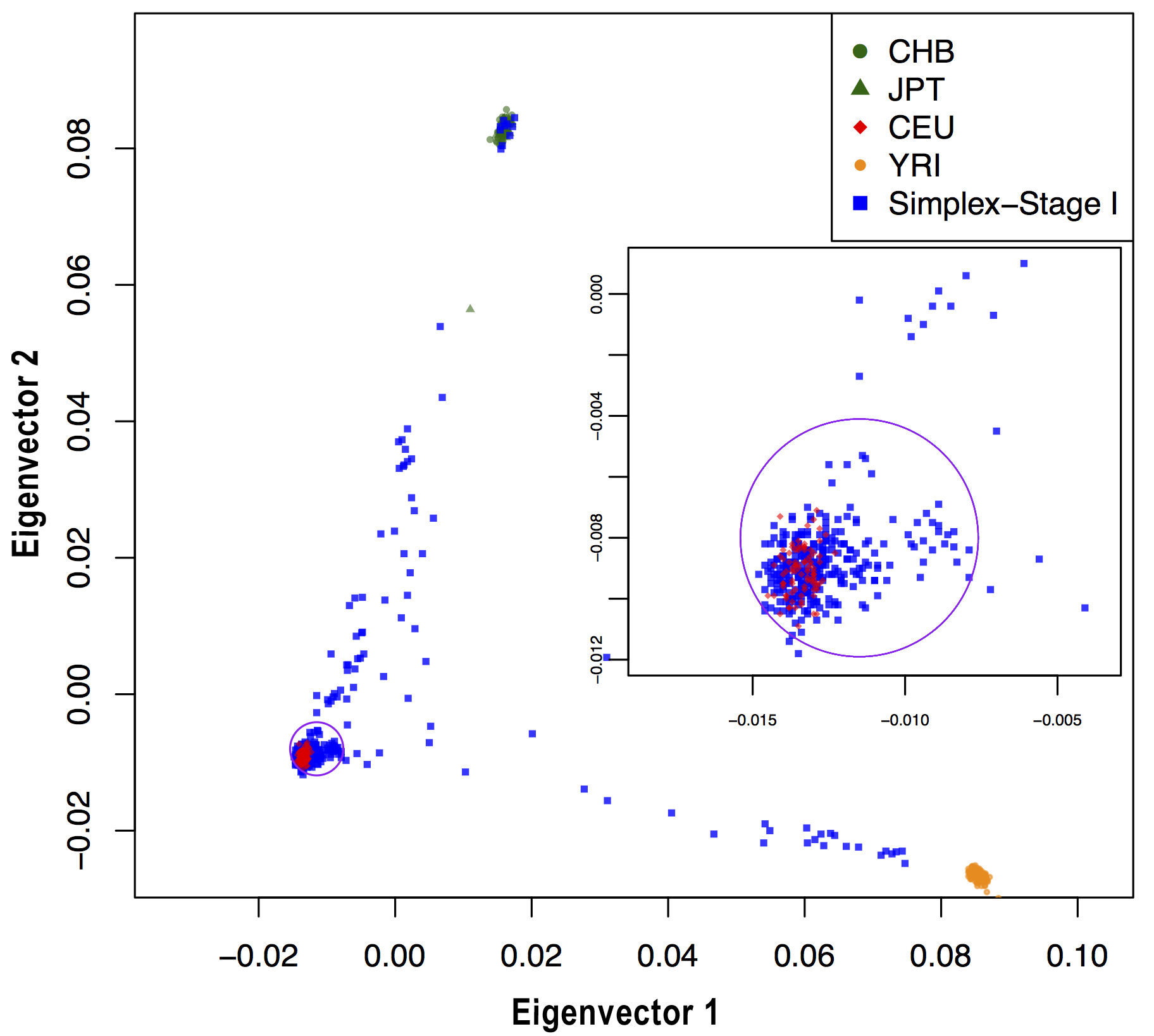


**Figure S3B**


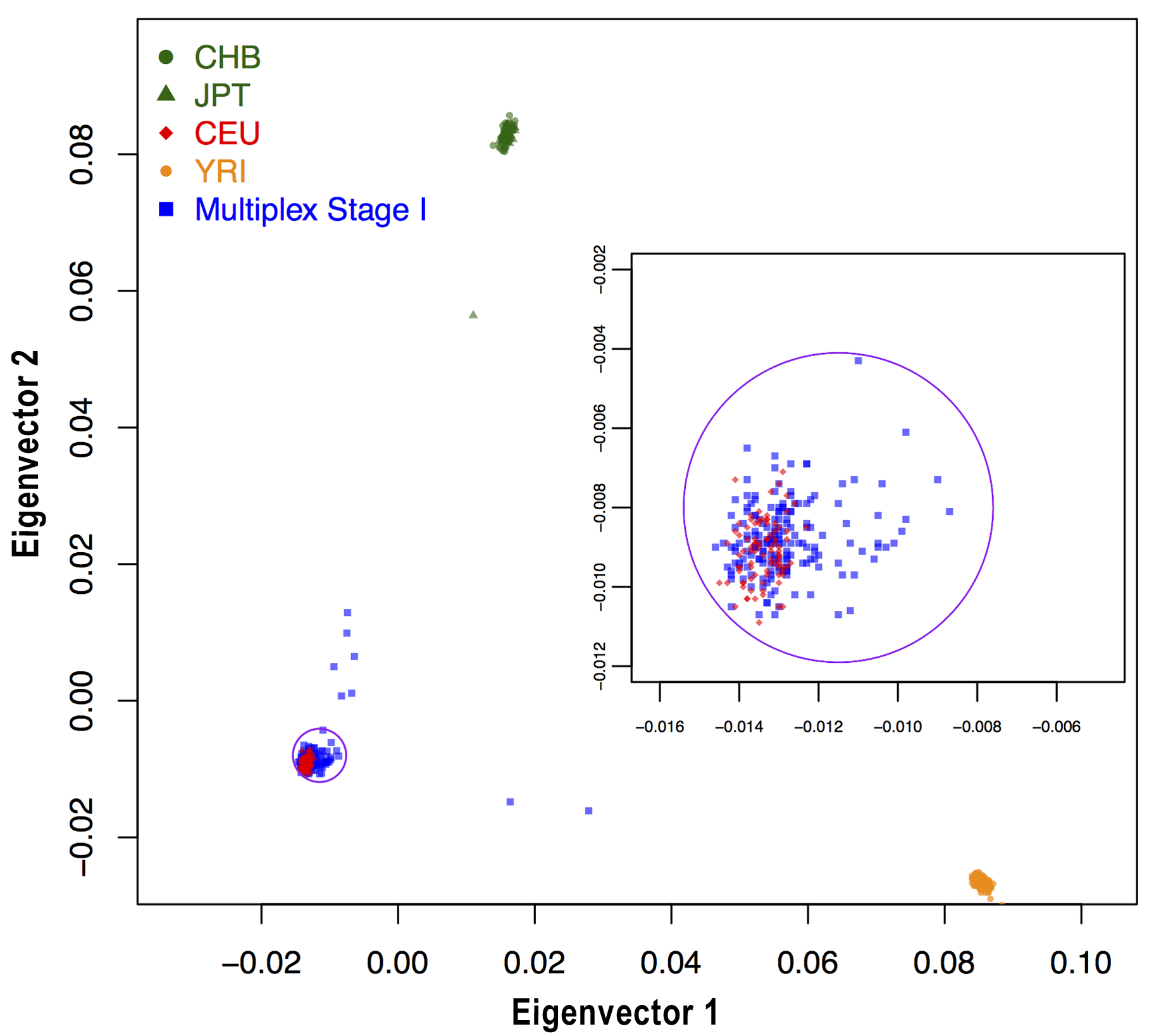


**Figure S3C**


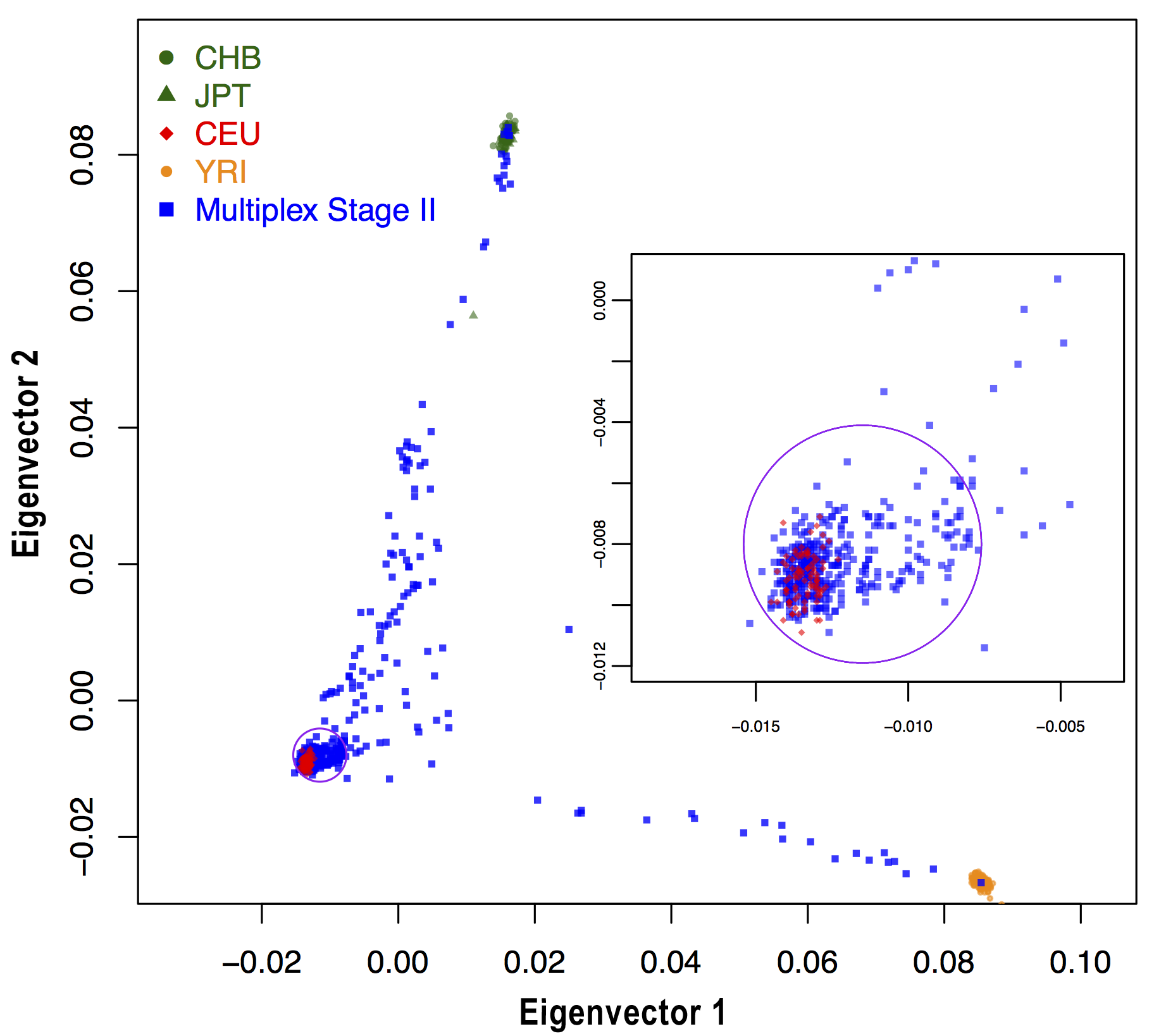


**Figure S4**


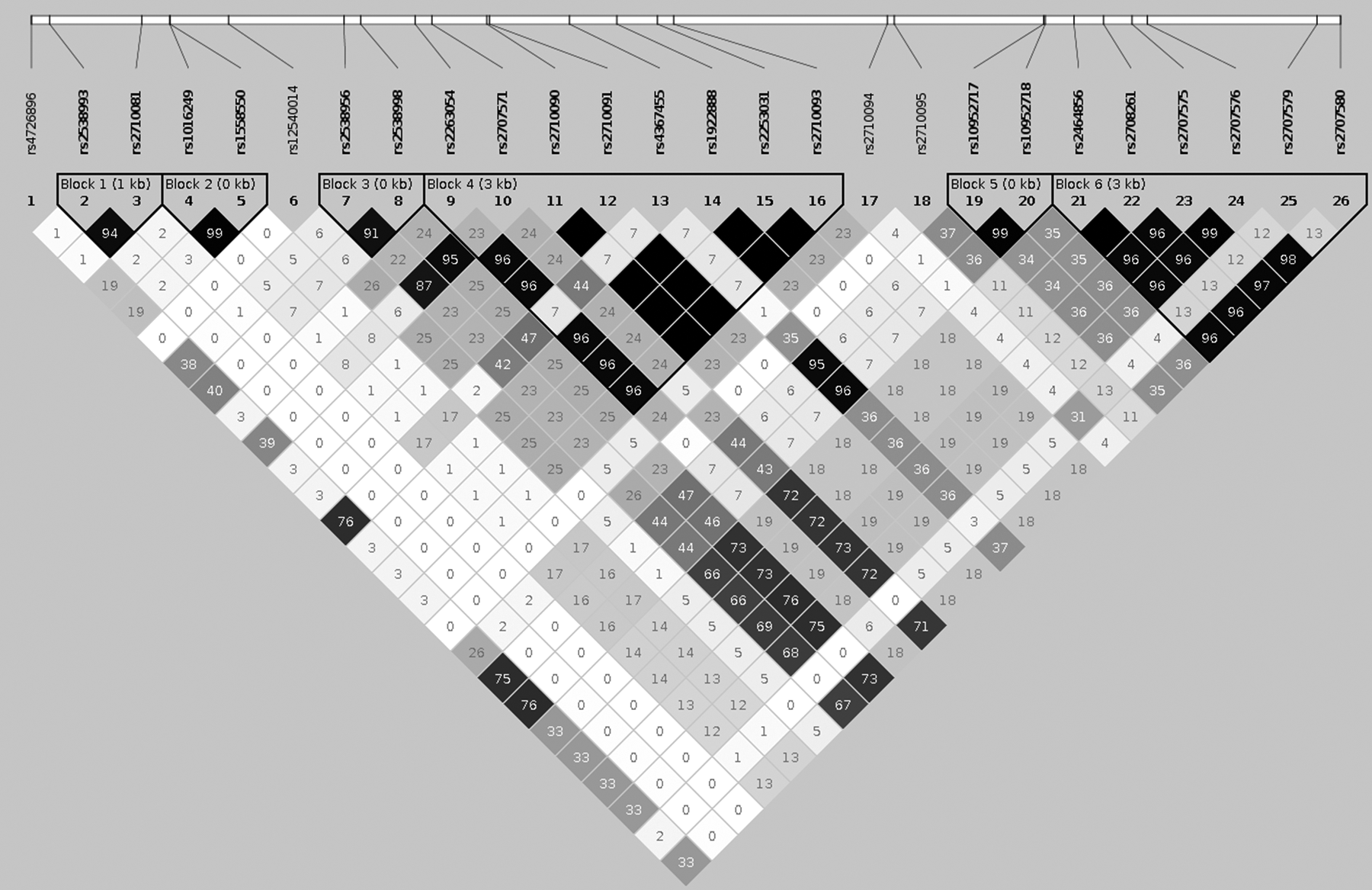


**Figure S5A**


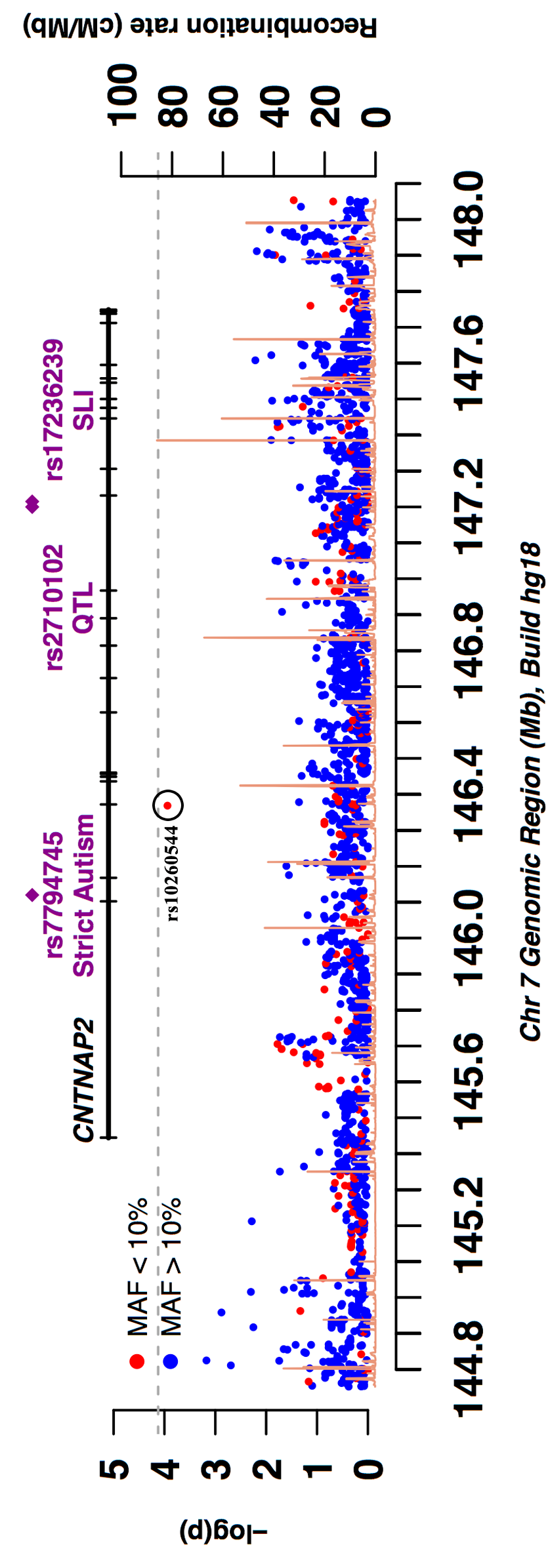


**Figure S5B**


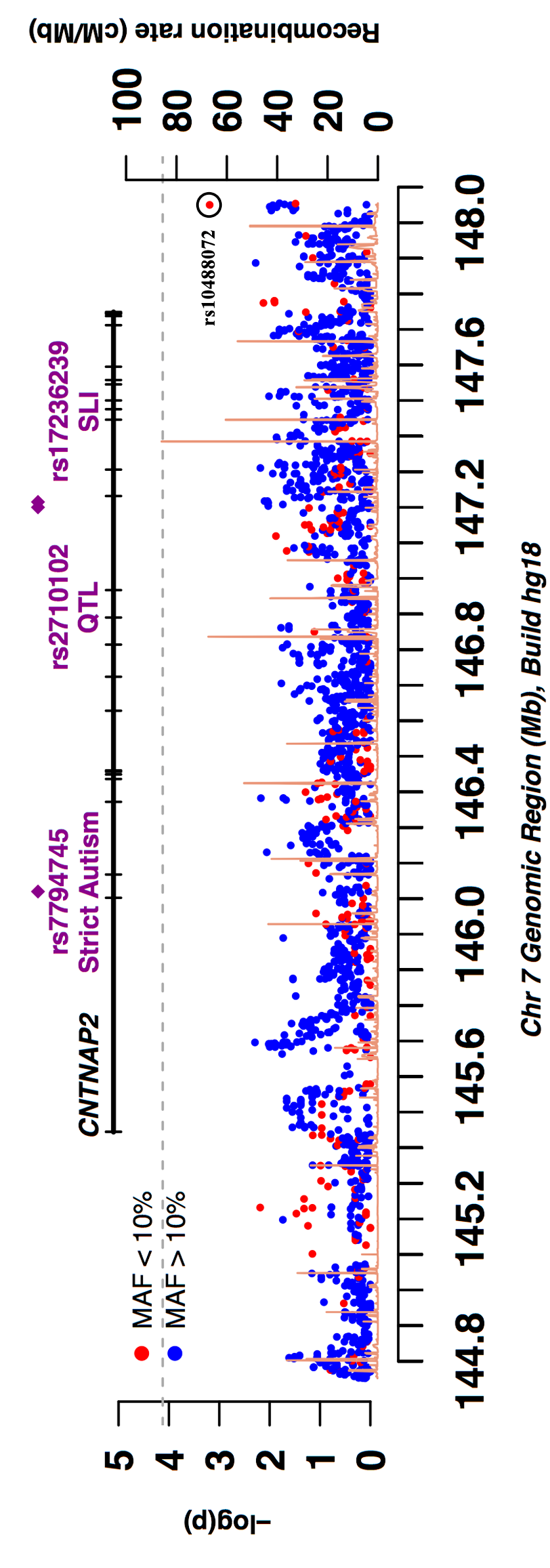

Supplement: Information S1 — Combined Supplementary Methods, Supplementary Tables and Supplementary Figures file. Additional details of the methods are provided in Supplementary Methods section. Supplemental tables and figures are provided in Supplementary Tables and Supplementary Figures section. (DOCX) [file pone.0077906.s001.docx]
